# The Combined Effects of Arbuscular Mycorrhizal Fungi (AMF) and Lead (Pb) Stress on Pb Accumulation, Plant Growth Parameters, Photosynthesis, and Antioxidant Enzymes in *Robinia pseudoacacia* L.

**DOI:** 10.1371/journal.pone.0145726

**Published:** 2015-12-23

**Authors:** Yurong Yang, Xiaozhen Han, Yan Liang, Amit Ghosh, Jie Chen, Ming Tang

**Affiliations:** 1 State Key Laboratory of Soil Erosion and Dryland Farming on the Loess Plateau, Northwest A&F University, Yangling, Shaanxi 712100, China; 2 College of Forestry, Northwest A&F University, Yangling, Shaanxi 712100, China; 3 Plant Systems Biology Lab, Botany and Plant Science, School of Natural Sciences, National University of Ireland, Galway, Ireland; 4 Joint BioEnergy Institute, 5885 Hollis St, Emeryville, California 94608, United States of America; 5 Physical Biosciences Division, Lawrence Berkeley National Laboratory, Berkeley, California 94720, United States of America; 6 School of Energy Science and Engineering, PK Sinha Centre for Bioenergy, Indian Institute of Technology Kharagpur, Kharagpur 721302, India; Sun Yat-Sen University, CHINA

## Abstract

Arbuscular mycorrhizal fungi (AMF) are considered as a potential biotechnological tool for improving phytostabilization efficiency and plant tolerance to heavy metal-contaminated soils. However, the mechanisms through which AMF help to alleviate metal toxicity in plants are still poorly understood. A greenhouse experiment was conducted to evaluate the effects of two AMF species (*Funneliformis mosseae* and *Rhizophagus intraradices*) on the growth, Pb accumulation, photosynthesis and antioxidant enzyme activities of a leguminous tree (*Robinia pseudoacacia* L.) at Pb addition levels of 0, 500, 1000 and 2000 mg kg^-1^ soil. AMF symbiosis decreased Pb concentrations in the leaves and promoted the accumulation of biomass as well as photosynthetic pigment contents. Mycorrhizal plants had higher gas exchange capacity, non-photochemistry efficiency, and photochemistry efficiency compared with non-mycorrhizal plants. The enzymatic activities of superoxide dismutase (SOD), ascorbate peroxidases (APX) and glutathione peroxidase (GPX) were enhanced, and hydrogen peroxide (H_2_O_2_) and malondialdehyde (MDA) contents were reduced in mycorrhizal plants. These findings suggested that AMF symbiosis could protect plants by alleviating cellular oxidative damage in response to Pb stress. Furthermore, mycorrhizal dependency on plants increased with increasing Pb stress levels, indicating that AMF inoculation likely played a more important role in plant Pb tolerance in heavily contaminated soils. Overall, both *F*. *mosseae* and *R*. *intraradices* were able to maintain efficient symbiosis with *R*. *pseudoacacia* in Pb polluted soils. AMF symbiosis can improve photosynthesis and reactive oxygen species (ROS) scavenging capabilities and decrease Pb concentrations in leaves to alleviate Pb toxicity in *R*. *pseudoacacia*. Our results suggest that the application of the two AMF species associated with *R*. *pseudoacacia* could be a promising strategy for enhancing the phytostabilization efficiency of Pb contaminated soils.

## Introduction

Soil contamination with toxic heavy metals (HMs) has become a critical environmental concern because of its potentially adverse effects on ecosystems. Elevated HMs can be attributed to a number of human activities and industrial processes such as mining, smelting, electroplating, coal burning, and the production of leaded gasoline [1]. Due to less advanced mining technologies and more emphasis on economic growth, the large amount of lead (Pb) derived from industrial activities has caused severe environmental pollution in China [2]. As one of the most abundant and non-essential elements in soil, high dose of Pb likely result in metabolic disorders, growth inhibition and even death for most plant species [3].

A high level of Pb in the chloroplasts may disturb chloroplast function by inhibiting the enzyme activities associated with chlorophyll biosynthesis, CO_2_ fixation, and the aggregation of the pigment protein complexes in photosystems [3]. Generally, photosystem II (PSII) is more sensitive to HM pollution and its function is inhibited to a much higher extent than that of PSI [4]. Further studies confirmed that HM exerts multiple deleterious effects on both the donor and acceptor sites of PSII, inhibiting the oxygen evolution activities and electron-transfer reactions [5]. However, HM exposure can induce the accumulation of reactive oxygen species (ROS) such as singlet oxygen $\left({\text{O}}_{2}^{\cdot -}\right)$, hydrogen peroxide (H_2_O_2_), and the hydroxyl radical (HO·), resulting in lipid peroxidation and in turn doing harm to the bio-function of cell membranes and plant growth [6,7]. Plants have developed many ways to alleviate HM toxicity and improve their ability to survive in HM polluted soil [8]. One way is to scavenge ROS by enhancing the activities of antioxidant enzymes, including superoxide dismutase (SOD), peroxidase (POD), catalase (CAT), ascorbate peroxidase (APX), glutathione peroxidase (GPX) and glutathione reductase (GR), to protect plant cells from disruption and dysfunction [8]. Another strategy is to establish symbiotic association in roots with arbuscular mycorrhizal fungi (AMF) to improve host HM resistance [9].

AMF are one of the most prominent soil microorganisms, engaging in mutual symbiosis with most terrestrial plants (70–90%) [10]. AMF can significantly enhance host plant HM tolerance by improving plant nutrient acquisition and by influencing the fate of the metals in both plants and soil [11]. The extraradical mycelium of AMF acts as a plant root extension and can reach beyond the root depletion zone, enabling a thorough exploration of the soil for water and mineral nutrients [12]. Therefore, the use of plants that can concentrate high amounts of HMs and in association with appropriate AMF species might be a promising method for the phytostabilization of HM polluted soils [2, 13]. However, the exact roles of AM association in host plant HM tolerance and phytostabilization efficiency are still largely unknown.

As one of the most important pioneer species, legumes can adapt to a wide range of environments, and they have been successfully employed for re-vegetation and restoration in arid and degraded ecosystems [14]. An increasing number of studies have indicated that *Robinia pseudoacacia* L. inoculated with appropriate AMF species could be an effective tool for phytostabilization of HM contaminated soils because of its fast growth, high accumulation of HMs, and atmospheric nitrogen fixation [2]. Two AMF species (*Funneliformis mosseae* and *Rhizophagus intraradices*) were selected on the basis of our previous investigation, which suggested their dominance in HM contaminated soil [15]. However, the response of plant growth, photosynthesis, and antioxidant enzymes to Pb toxicity remains controversial and can vary depending on the plant species, exposure duration, and stress levels [16]. Moreover, little information is available about the exact roles of AMF in host physiological and biochemical responses to Pb exposure, although it is well-known that AMF symbiosis has the ability to improve plant growth and HM tolerance [17]. In addition, the effects of short- or long-term HM exposure on photosynthetic activity can be reflected by chlorophyll fluorescence parameters, which have been widely used as an informative tool for studying the electron transport chain associated with photosystem II (PSII) in various plant species [18]. The purpose of the present study was to determine the effects of AM association and Pb stress (0, 500, 1000, and 2000 mg kg^-1^) on the growth, Pb accumulation, gas exchange, chlorophyll fluorescence and antioxidant enzymes of *R*. *pseudoacacia* to better understand the mechanisms of Pb alleviation and phytostabilization caused by AMF. Our study could provide experimental evidence for using these leguminous tree (*R*. *pseudoacacia*) in symbiotic association with appropriate AMF species for the phytostabilization and ecological restoration of Pb polluted soils.

## Materials and Methods

### Plant and AM inocula

The seeds of *R*. *pseudoacacia* were collected from HM polluted area located in Feng County (106°39′26″E, 33°51′21″N), Shaanxi Province in 2011, and then they were stored in freezer (4°C) until ready to use. The area is not privately-owned or protected in any way, so no specific permits were required for the seed collection. The area did not involve endangered or protected species. In November, 2012, The seeds were surface-sterilized in 10% (v/v) sodium hypochlorite for 10 min, washed five times with sterile water (floating seeds were discarded), and then pre-germinated on sterile gauze in petri dishes at 25°C in dark. After three days, two geminated seeds were transferred to plastic pot. One week later, only one seedling of uniform growth was kept and left growing for 65 subsequent days prior to harvest.

Two AMF species, *Funneliformis mosseae* (T.H. Nicolson & Gerd.) C. Walker & A. Schüßler (BGC GZ01A) and *Rhizophagus intraradices* (N.C. Schenck & G.S. Sm.) C. Walker & A. Schüßler (BGC BJ09), were obtained from Beijing Academy of Agriculture and Forestry Sciences, Beijing, P.R. China (http://www.yzs.baafs.net.cn/Default.aspx). They were multiplied in pot culture for 4 months in greenhouse conditions using *Zea mays* (L.). The average colonization of *F*. *mosseae* and *R*. *intraradices* were 94.2 and 96.8% and average spore density were 619 and 549 per 10 g of air-dried soil, respectively. Spores, mycelium, colonized root fragments, and dried sand-soil were mixed to use as AMF inocula according to our previous study [14]. In the non-mycorrhizal treatment, 10 mL of the filtered leachate was added in each pot (15 μm sieve) to compensate for the free-living microbiota associated with the mycorrhizal inoculum.

### Soil preparation and experimental design

The culture medium consisted of soil and sand (1:3, v/v). Soil was collected from the top layer (0–15 cm) in the Nursery of Forestry College, Northwest A&F University in Yangling, Shaanxi Province, China, and sieved with a 2-mm diameter sieve. The basic physical and chemical properties of the soil were described as follows: pH 7.8 (soil: water, 1:5), 17.4 g kg^-1^ of soil organic matter, 2.18 g kg^-1^ of total nitrogen, 45.4 mg kg^-1^ of available nitrogen, 1.13 g kg^-1^ of total phosphorus, 31.7 mg kg^-1^ of available phosphorus, 179.2 mg kg^-1^ of available potassium, 17.9 mg kg^-1^ of total Pb. The soil substrate was mixed with fine sand and autoclaved under pressure (0.11 MPa) at 121°C for 2 h to eliminate all microorganisms. Each plastic pot was filled with 1.5 kg of medium mixture and 10 g AM inoculums (placed 1–2 cm below plants). All pots were well watered and Hoagland’s nutrient solution [19] was applied every three weeks throughout the growth period of plants.

The experiment was conducted in the greenhouse of Northwest A&F University in Shaanxi province of China (34°15'59"N, 108°03'39"E) from November 2012 to January 2013. The average temperature was 20–30°C and the relative air humidity was 50–75%. The experiment was a 4 × 3 complete factorial design which was comprised of four Pb addition levels and three inoculation treatments with twenty replicates for each treatment. It was carried out with the following treatments: non-mycorrhizal control and inoculation with *F*. *mosseae* or *R*. *intraradices*, each at four Pb stress levels of 0, 500, 1000, and 2000 mg kg^-1^ soil. In all, two hundred and forty pots were arranged in a randomized complete block design.

### Mycorrhizal colonization

Measurements were made 75 days after the experiment started, the root samples of plants were collected, washed with tap water, and then cut into about 1-cm length segments. The segments were first softened in 2.5% KOH at 90°C for 1 h, bleached in alkaline hydrogen peroxide at room temperature for 30 min, acidified in 1% HCl at room temperature for 1 h, and then stained with trypan blue (0.05%) at 90°C for 20 min. The mycorrhizal colonization (MC) was estimated according to Trouvelot et al [20]. At least 100 root fragments per treatment were used to measure the MC under a light microscope (Olympus Bx51, Japan) at 200 × magnification. The percentage of mycorrhizal structures in each 1-cm root fragment was assessed as 0, 10, 20…100%. The MC was calculated according to Eq 1:

$$\text{MC\%}=\frac{\sum \left(0\text{\%}\times {\text{N}}_{0}+10\text{\%}\times {\text{N}}_{10}+20\text{\%}\times {\text{N}}_{20}+\ldots +100\text{\%}\times {\text{N}}_{100}\right)}{\left({\text{N}}_{0}+{\text{N}}_{10}+{\text{N}}_{20}+\ldots +{\text{N}}_{100}\right)}$$ (1)

Where N is the number of root fragments.

### Plant growth parameters

Plant height and stem diameter were measured by using a precision straight edge (Sword fish, China). Plant leaves, stems, and roots were washed separately with tap water, and then dried in an oven at 80°C for 48 h to determine dry biomass [21].

### Photosynthetic parameters

Net photosynthetic rate (Pn), stomatal conductance (g_s_), intercellular CO_2_ concentration (C_i_), and transpiration rate (T_r_) were measured using a portable open flow gas exchange system LI-6400 (LI-COR, USA) from 9:00 a.m. to 11:00 a.m. on the fifth youngest leaf [17]. The photosynthetically active radiation was 1000 ± 17 μmol m^-2^ S^-1^; the leaf temperature was 26.0 ± 1.0°C; CO_2_ concentration and the ambient water vapor pressure were kept at 480 ± 10 cm^3^ m^-3^ and 1.35 ± 0.10 kPa, respectively [14]. The ratio of net photosynthetic rate per transpiration rate was used to measure water use efficiency (WUE).

### Chlorophyll fluorescence parameters

Chlorophyll fluorescence parameters were measured using a MINI-Imaging-PAM (Walz, Germany) according to the method described in our previous study [22]. Briefly, the fifth fully expanded leaf of each seedling was placed in the dark for 30 min and then the minimal fluorescence in the dark-adapted state (F0) was recorded at room temperature. A saturating pulse of irradiation 2000 μmol m^-2^ s^-1^ was applied for 3 s to determine the maximal fluorescence (Fm) in the dark-adapted state. The leaves were then illuminated with actinic light (300 μmol m^-2^ s^-1^) for 10 min in order to measure minimum fluorescence (F_0_′), maximal fluorescence (F_m_′), and steady-state fluorescence (F_s_). The maximal quantum yield of PSII in the dark-adapted state (Fv/Fm), photochemical quenching coefficient (qP), non-photochemical quenching coefficient (qN), and effective photochemical efficiency of PSII (ΦPSII) were calculated using the following equations: Fv/Fm = (Fm − F0)/Fm; qP = (F_m_′ − Fs)/(F_m_′ − F_0_′); qN = (Fm − F_m_′)/ F_m_′; ΦPSII = (F_m_′ − Fs)/F_m_′.

### Photosynthetic pigment

The contents of chlorophyll (Chl) *a*, Chl *b* and total carotenoids (Car) were calculated according to the method described by Gao [21]. Fresh leaf samples (the fifth leaf) were cleaned with deionized water to remove any surface contamination and 100 mg fresh sample was homogenized in 25 mL acetone (80%) in the dark at room temperature for 10 h. A UV/Vis spectrophotometer (UV-1800, Shimadzu, Japan) was used to measure the content of photosynthetic pigment at 646, 663 and 470 nm. The contents of chlorophyll and carotenoids were calculated using the following equations:

$$\text{Chl }a\;\left({\text{mg g}}^{-1}\right)=\left(12.21\;\times {\text{A}}_{663}-2.81\times {\text{A}}_{646}\right)/\text{FW}$$ (2)

$$\text{Chl }b\;\left({\text{mg g}}^{-1}\right)=\left(20.13\;\times {\text{A}}_{646}-5.03\times {\text{A}}_{663}\right)/\text{FW}$$ (3)

$$\text{Car }\left({\text{mg g}}^{-1}\right)=\frac{1000\;\times {\text{ A}}_{470}\;-\;3.27\;\times \text{ Chl }a\;-\;104\;\times \text{ Chl }b}{229\;\times \;\text{FW}}$$ (4)

Where A_λ_ = absorbance at λ (nm); FW, fresh weight of leaf samples.

### Enzymes extraction and assays

The leaves of black locust were collected and washed with distilled water, and then the surface moisture was wiped out. For enzyme extracts and assays, the fresh leaf samples (0.5 g) were frozen in liquid nitrogen and then grounded into a fine powder with a pre-chilled mortar and pestle in 5 mL of ice cold 50 mM potassium phosphate buffer (pH 7.0), containing 1 mM EDTA, 2 mM ascorbic acid (ASA) and 1% (w/v) soluble polyvinylpolypyrrolidone (PVPP). After filtration (Millipore, Mitex 0.5 mm), the homogenate was centrifuged at 12000 g for 20 min and the supernatant was collected and used for SOD, POD, CAT, APX and GR assays. All operations were done at 4°C.

SOD activity was assayed by measuring its ability to inhibit the photochemical reduction of NBT according to the method described by Gao [21]. The reaction buffer (2.8 mL) contained 50 mM potassium phosphate buffer (pH 7.8), 0.1 mM EDTA, 130 mM methionine, 0.75 mM NBT, 20 μM riboflavin and 0 or 100 μL of the enzyme extract. The reaction was started by adding riboflavin (20 μM) and the tubes were shaken and placed 30 cm below a light bank consisting of two 15 W fluorescent lamps for 20 min. Reaction was stopped by switching off the light and wrapping the tubes with black cloth. The absorbance of irradiated and non-irradiated (blank) solution was measured at 560 nm. One unit of SOD activity was taken as the quantity of enzyme which reduced the absorbance reading of samples to 50% in comparison with tubes lacking enzymes. SOD activity was expressed as U g^-1^ FW h^-1^.

CAT activity was assayed by measuring the rate of disappearance of hydrogen peroxide (H_2_O_2_) according to the method described by Gao [21]. The reaction buffer (3.0 mL) contained 50 mM phosphate buffer (pH 7.0), 200 mM hydrogen peroxide (H_2_O_2_), and 100 μL enzyme extracts. Decrease in hydrogen peroxide was measured as the decline in optical density at 240 nm for 1 min. One unit of CAT activity was taken as the decrease of absorbance density at 240 nm by 0.1 unit per minute. CAT activity was expressed as U g^-1^ FW min^-1^.

POD activity was measured as the change of absorbance of 470 nm due to guaiacol oxidation according to the method described by Gao [21]. The reaction buffer (10 mL) contained 10 mM potassium phosphate buffer (pH 7.0), 1% (w/v) guaiacol and 100 μL of enzyme extract. The reaction was initiated with adding 1.0 mL of 0.18% (w/v) H_2_O_2_. The oxidation of guaiacol in the presence of H_2_O_2_ was measured at 470 nm for 1 min. One unit of peroxidase activity was defined as the quantity of 1% (w/v) guaiacol oxidized per minute. POD activity was expressed as U g^-1^ FW min^-1^.

APX activity was estimated according to the method described by Nakano and Asada [23]. The reaction buffer (1.0 mL) contained 50 mM phosphate buffer (pH 7.0), 0.5 mM ascorbate, 0.1 mM EDTA, 1.0 mM H_2_O_2_ and 100 μL enzyme extract. The reaction was started by addition of H_2_O_2_ and ascorbate oxidation measured at 290 nm (extinction coefficient 2.8 mM^-1^ cm^-1^) for 1 min. One unit of APX was defined as the amount of the enzyme required to oxidize 1 μmol ascorbate per mg of soluble protein per min. The enzyme activity was expressed as U g^-1^ FW min^-1^.

GPX activity was assayed according to the method described by Hemeda and Klein [24]. A 100 mL of reaction mixture contained 10 mL of 1% guaiacol (v/v), 10 mL of 0.3% H_2_O_2_ and 80 mL of 50 mM phosphate buffer (pH 6.6). 75 μL of enzyme extract was added to reaction mixture with a final volume of 3 mL. An increase in absorbance due to guaiacol oxidation was monitored at 470 nm for 3 min and enzyme activity was expressed as U g^-1^ FW min^-1^.

GR activity was assayed as described by Foyer and Halliwell [25]. The reaction buffer (1.0 mL) consisted of 25 mM phosphate buffer (pH 7.8), 0.1 mM EDTA, 0.2 mM NADPH, 0.5 mM oxidized glutathione (GSSG) and 100 μL enzyme extract. The reaction was started by the addition of GSSG and enzyme activity was determined using the molar extinction coefficient (6.2 mM^-1^ cm^-1^) for NADPH. One unit of GR activity was defined as 1.0 g tissue proteins consumed 1μmol NADPH at 340 nm for 1 min. GR activity was expressed as U g^-1^ protein min^-1^.

### Hydrogen peroxide and lipid peroxidation contents

The content of H_2_O_2_ was determined according to the method described by Patterson et al [26] with minor modifications. The fresh leaf samples of black locust (500 mg) were homogenized in 5 mL ice cold acetone and centrifuged at 10000 g at 4°C for 10 min. Titanium sulphate (TiS_2_O_8_; 5%, w/v) and ammonia were added to supernatant and centrifuged at 10000 g at 4°C for 10 min. The precipitate was washed five times with ice acetone by resuspension, drained, and dissolved in 3 mL of 10% (v/v) H_2_SO_4_. The reaction was monitored by the absorption of titanium peroxide complex at 410 nm with a UV/Vis spectrophotometer (UV-1800, Shimadzu, Japan) according to a standard curve. The content of H_2_O_2_ was expressed as μmol g^-1^ FW.

The level of MDA has been routinely used as an index for the status of lipid peroxidation under stress conditions. MDA content was estimated using thiobarbituric acid (TBA) method as described by Heath and Packer [27]. The fresh leaf samples (500 mg) were grounded into a fine powder using a pre-chilled mortar and pestle in 5 mL buffer containing 10% trichloroacetic acid (TCA) and 0.25% 2-thiobarbituric acid. The homogenate was incubated in boiling water bath for 30 min and reaction was stopped in ice, and then the samples were centrifuged at 10000 g for 10 min. The concentration of MDA was calculated from the absorbance at 532 nm and corrected for unspecific turbidity by subtracting the absorbance of the same at 600 nm using extinction coefficient of 155 mM^-1^ cm^-1^. The level of MDA was expressed as nmol g^-1^ FW.

### Statistical analysis

The translocation factor indicates the efficiency of a plant translocate the metal from its root to aerial parts [28]. It is calculated according to Eq 5:

$$\text{Translocation Factor }\left(\text{TF}\right)=\;\frac{{\text{C}}_{\text{Leaf}}}{{\text{C}}_{\text{Root}}}$$ (5)

Where C_Leaf_ and C_Root_ are the Pb concentration in plant leaf and root, respectively.

The mycorrhizal dependency (MD) presents the degree to which a plant relies upon the mycorrhizal condition to produce its maximum growth at a given level of soil fertility [29]. It is calculated according to Eq 6:

$$\text{MD}=\frac{{\text{DW}}_{\text{AMF}}-{\text{DW}}_{\text{NM}}}{{\text{DW}}_{\text{AMF}}}$$ (6)

Where DW_AMF_ is the total dry weight of the AMF inoculated plants and DW_NM_ is the total dry weight of the non-inoculated plants.

Prior to data analysis, the Kolmogorov-Smirnov test was used to check for data normality and the Levene test for homogeneity of variance in SPSS 16.0 (SPSS Inc., Chicago, IL, USA) for Windows 7. In the present study, all the original datasets conformed to a normal distribution. When necessary, dependent variables were transformed using the natural logarithmic, arcsine or Box-Cox functions to achieve requirements of homogeneity of variance (P > 0.05). Potential differences among different treatments were analyzed using two-way and three-way ANOVAs followed by Duncan’s multiple range tests. Six plants randomly selected were analyzed per treatment. Experimental data were subjected to one-way and two-way ANOVAs, and the means were compared by Duncan's multiple-range tests, P < 0.05 (SPSS software version 16.0). One-way ANOVAs were used to analyze the significance of the effects of Pb addition on MC, plant dry weight, shoot height, Pb concentrations in different tissues, contents of photosynthetic pigments, chlorophyll fluorescence and gas exchange parameters, activities of antioxidant enzymes and MD of *R*. *pseudoacacia*. Two-way ANOVAs were used to determine the significance of the effects of Pb addition, AMF inoculation and their interactions on plant dry weight, shoot height, Pb concentrations in different tissues, contents of photosynthetic pigments, chlorophyll fluorescence and gas exchange parameters, and activities of antioxidant enzymes of *R*. *pseudoacacia*.

## Results

### Mycorrhizal colonization

**Fig 1:**
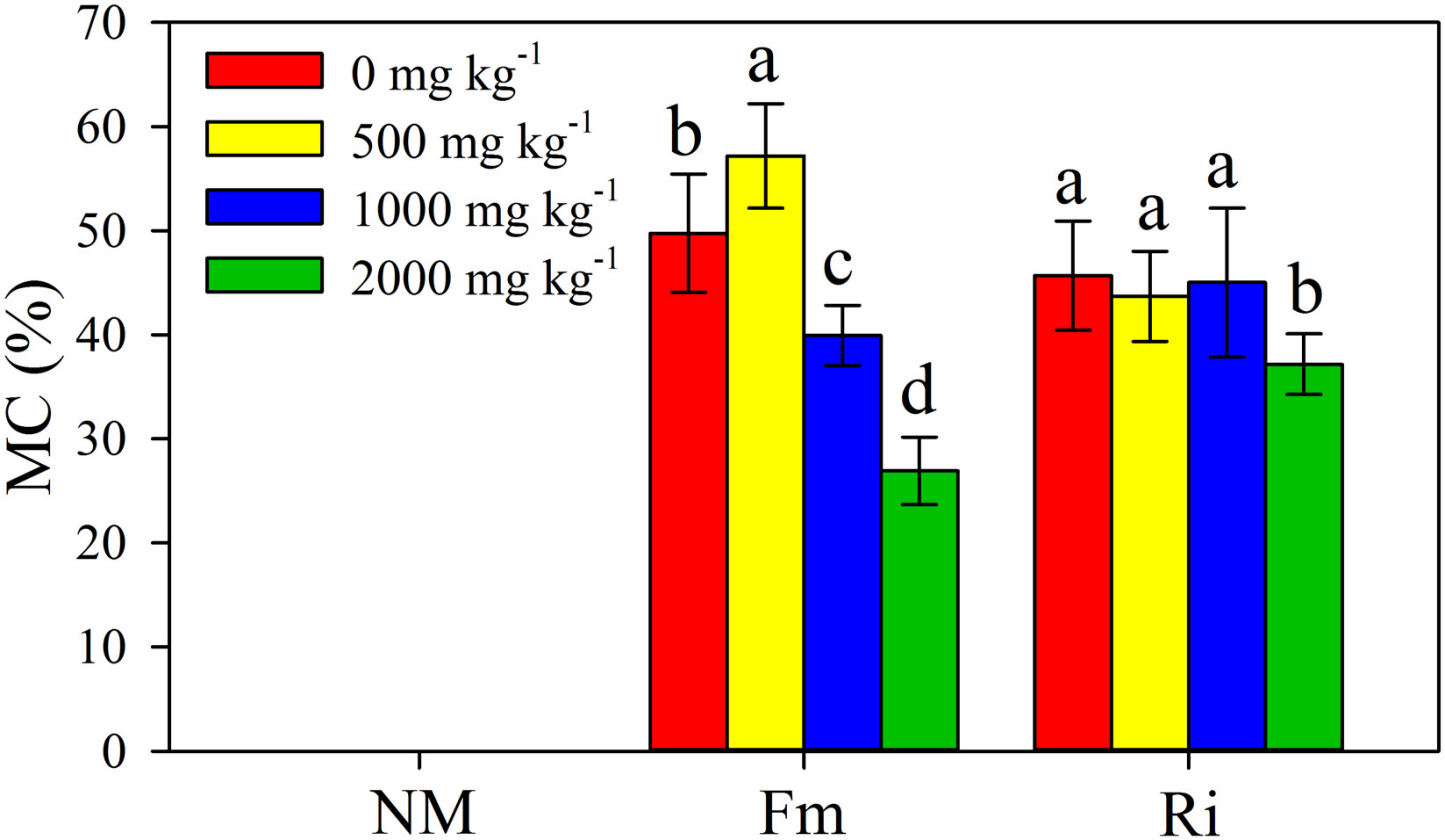
Mycorrhizal colonization (MC) of *R*. *pseudoacacia* was affected by Pb stress levels. NM, non-inoculated control; Fm, inoculated with *F*. *mosseae*; and Ri, inoculated with *R*. *intraradices*. The results are reported as the mean (n = 6) ± SD. The same letter indicates no significant difference among treatments (Duncan’s test, P < 0.05). Two-way ANOVAs were used to determine the significance of the effects of the Pb level (Pb), AMF inoculation (AMF), and their interactions (Pb × AMF) on the MC of black locust seedlings, as shown in S1 Table.

A microscopic assessment confirmed a good symbiotic association between AMF and plant roots (Fig 1). Under control treatments, almost no MC could be detected in the roots of non-mycorrhizal plants (< 1%). The residue level of the MC (< 1%) was likely to be a false positive (a small amount of stain was randomly retained by the root hairs, vessel cells, etc.) when scoring root slides. The MC of *F*. *mosseae* (Fm) plants first increased and then decreased with increasing Pb stress levels, and the highest MC (57%) was detected at the 500 mg kg^-1^ Pb stress level. However, no difference in the MC of *R*. *intraradices* (Ri) plants could be found within the range of Pb concentrations (0, 500, and 1000 mg kg^-1^), and the lowest MC (37%) was observed at the highest Pb stress level (2000 mg kg^-1^). It is interesting to note that the Ri plants had much larger MC compared with that of Fm plants in the 2000 mg kg^-1^ Pb treatment (P < 0.05).

### Plant growth parameters

Pb addition significantly decreased the shoot height and the leaf, stem and root dry weights of both mycorrhizal and non-mycorrhizal plants (Table 1). By contrast, the mycorrhizal plants had much higher shoot heights and leaf, stem and root dry weights than non-mycorrhizal plants in all treatments, with the exception of the root dry weight in the control (0 mg kg^-1^ Pb). No significant difference in the shoot heights and leaf, stem and the root dry weights were detected between plants that were inoculated with *F*. *mosseae* or *R*. *intraradices* at 0, 500 and 1000 mg kg^-1^ Pb stress levels, whereas the plant heights and dry weights (leaf, stem and root) of Ri plants were significantly higher than those of Fm plants at the highest Pb stress level (2000 mg kg^-1^).

**Table 1:** The shoot height, the dry weights of the leaves, stems, roots and the total dry weight of *Robinia pseudoacacia* were affected by Pb stress levels and AMF inoculation.

Pb level (mg kg^-1^)	AMF inoculation	Shoot height (cm)	Dry weight (g)				
			Leaf	Stem	Shoot	Root	Total
0	NM	37.9±3.16b	3.76±0.33b	1.50±0.16b	5.26±0.32b	2.57±0.25a	7.83±0.51b
	Fm	45.5±3.40a	4.82±0.45a	1.71±0.08a	6.53±0.52a	2.26±0.17b	8.79±0.63a
	Ri	43.6±3.14a	4.58±0.33a	1.76±0.18a	6.34±0.34a	2.16±0.06b	8.50±0.39a
500	NM	35.3±3.30b	3.21±0.28b	1.47±0.14a	4.67±0.41b	1.89±0.09a	6.56±0.35b
	Fm	43.9±5.87a	4.41±0.37a	1.71±0.20a	6.12±0.41a	2.16±0.11b	8.28±0.42a
	Ri	39.8±3.47ab	4.57±0.27a	1.62±0.28a	6.20±0.34a	2.17±0.12b	8.37±0.39a
1000	NM	20.3±2.37b	2.38±0.27c	0.98±0.08b	3.36±0.30c	1.47±0.13b	4.83±0.33c
	Fm	31.3±2.95a	3.43±0.31b	1.31±0.15a	4.74±0.28b	1.81±0.11a	6.55±0.34b
	Ri	34.1±2.16a	3.96±0.39a	1.42±0.20a	5.38±0.31a	1.95±0.15a	7.33±0.43a
2000	NM	16.6±2.71c	1.98±0.19c	0.96±0.09c	2.94±0.22c	1.24±0.08c	4.18±0.20c
	Fm	23.8±2.22b	2.93±0.30b	1.21±0.13b	4.13±0.32b	1.59±0.11b	5.73±0.33b
	Ri	28.1±3.36a	3.35±0.22a	1.45±0.13a	4.80±0.31a	1.72±0.10a	6.52±0.31a
Significance							
Pb		0.00**	0.00**	0.00**	0.00**	0.00**	0.00**
AMF		0.00**	0.00**	0.00**	0.00**	0.00**	0.00**
Pb × AMF		0.005**	0.047*	0.12ns	0.02*	0.00**	0.00**

NM, non-inoculated control; Fm, inoculated with *F*. *mosseae*; and Ri, inoculated with *R*. *intraradices*. The results are reported as the mean (n = 6) ± SD. The same letter within each Pb level indicates no significant difference (P < 0.05).

** P < 0.01;

* P < 0.05; ns, no significance.

### Pb accumulation

**Fig 2:**
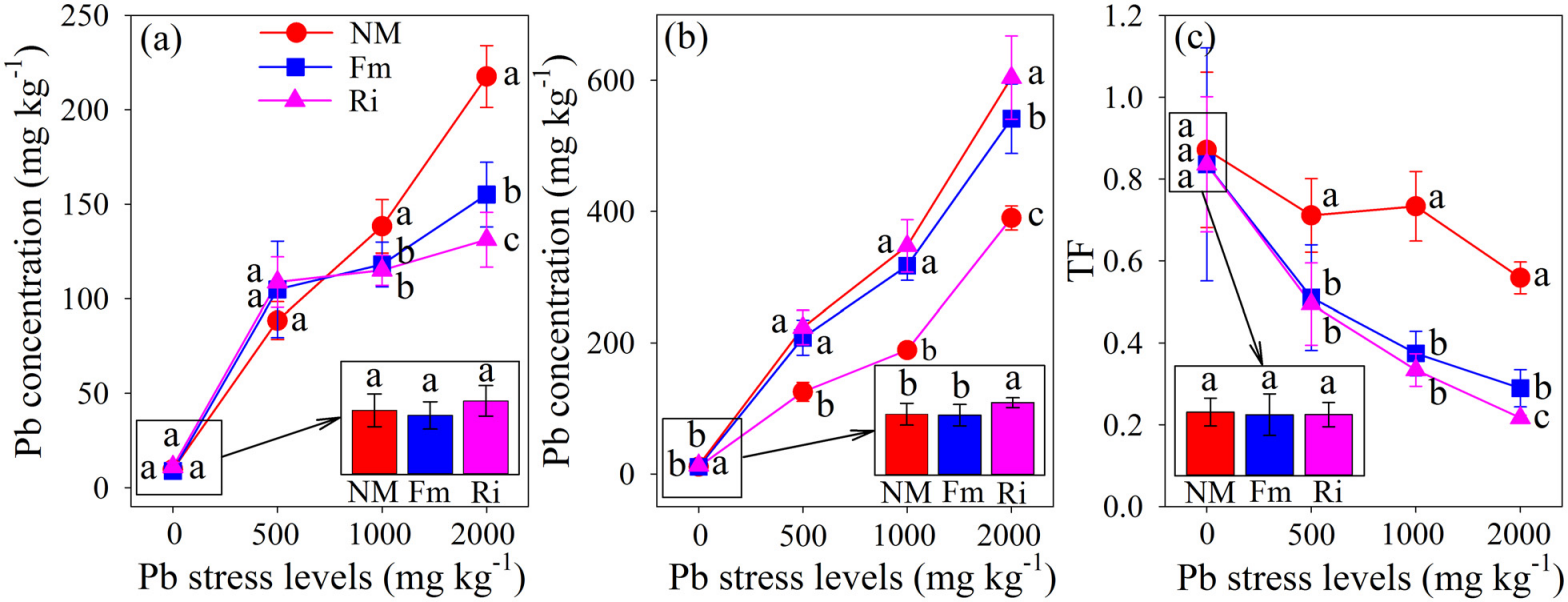
Pb concentrations in leaves (a) and roots (b), and the TF (c) of *R*. *pseudoacacia* were affected by Pb stress levels and AMF inoculation. NM, non-inoculated control; Fm, inoculated with *F*. *mosseae*; and Ri, inoculated with *R*. *intraradices*. The results are the mean (n = 6) ± SD. The same letter indicates no significant difference among treatments (Duncan’s test, P < 0.05). Two-way ANOVAs were used to determine the significance of the effects of Pb stress (Pb), AMF inoculation (AMF), and their interactions (Pb × AMF) on the Pb concentration and TF of black locust seedlings, as shown in S1 Table.

The Pb concentrations of leaves and roots of mycorrhizal and non-mycorrhizal plants are shown in Fig 2, and the Pb concentrations increased with increasing Pb stress levels in the soil. The leaves of the mycorrhizal plants had lower Pb concentrations than the leaves of non-mycorrhizal plants from 1000 and 2000 mg kg^-1^ Pb, but no difference in the leaf Pb concentration could be detected between Fm and Ri plants (Fig 2a). However, the Pb concentration in the roots of mycorrhizal plants was significantly higher than it was in the roots from non-mycorrhizal plants in all treatments except the control (0 mg kg^-1^ Pb) (Fig 2b). The TF of Pb in mycorrhizal plants was significantly reduced with increasing Pb concentration in the soil; however, the TF of Pb in non-mycorrhizal plants did not show significant differences among the first three Pb treatments (0, 500 and 1000 mg kg^-1^). The lowest TF values in mycorrhizal and non-mycorrhizal plants were found at the highest Pb stress level (2000 mg kg^-1^).

### Photosynthetic pigments

The Chl *a* and total Chl contents, and the Chl *a*/*b* ratio of *R*. *pseudoacacia* significantly decreased with increasing Pb stress levels, and mycorrhizal plants had much higher Chl *a*, total Chl contents and Chl *a*/*b* ratio compared with those of non-mycorrhizal plants (Table 2). However, no effects from Pb addition and AMF inoculation could be found on the Chl *b* content and Car/Chl ratio in the leaves. It is interesting to note that Pb addition significantly reduced the Car content of non-mycorrhizal plants in all treatments, but it did not influence that of mycorrhizal plants. The Car contents of Fm and Ri plants did not show considerable differences in all the treatments.

**Table 2:** Leaf contents of total chlorophyll (Chl), Chl *a*, Chl *b*, carotenoid (Car), and the Chl *a*/*b* and Car/Chl ratios of *R*. *pseudoacacia* were affected by Pb stress levels and AMF inoculation.

Pb level (mg kg^-1^)	AMF inoculation	Chlorophyll content (mg g^-1^)			Car (mg g^-1^)	Ratio	
		*a*	*b*	total		Chl *a*/*b*	Car/Chl
0	NM	1.51±0.14a	0.76±0.11a	2.26±0.18a	0.48±0.03b	2.03±0.35a	0.21±0.03a
	Fm	1.56±0.17a	0.83±0.09a	2.39±0.24a	0.56±0.03a	1.89±0.15a	0.23±0.02a
	Ri	1.55±0.12a	0.85±0.05a	2.40±0.15a	0.56±0.03a	1.82±0.11a	0.24±0.01a
500	NM	1.46±0.12a	0.64±0.07b	2.10±0.10b	0.46±0.04b	2.30±0.44a	0.22±0.02a
	Fm	1.58±0.16a	0.73±0.09ab	2.31±0.21ab	0.53±0.05a	2.17±0.27a	0.23±0.04a
	Ri	1.65±0.20a	0.77±0.11a	2.42±0.22a	0.54±0.01a	2.18±0.42a	0.23±0.02a
1000	NM	1.42±0.13a	0.57±0.04b	1.99±0.16b	0.41±0.03b	2.49±0.20a	0.21±0.01a
	Fm	1.49±0.14a	0.69±0.08a	2.19±0.12a	0.49±0.03a	2.19±0.38a	0.23±0.02a
	Ri	1.58±0.14a	0.66±0.04a	2.24±0.15a	0.50±0.04a	2.41±0.25a	0.22±0.03a
2000	NM	1.18±0.17b	0.47±0.04b	1.65±0.21b	0.37±0.02b	2.52±0.13a	0.23±0.03a
	Fm	1.45±0.11a	0.59±0.05a	2.04±0.11a	0.48±0.05a	2.48±0.33a	0.23±0.02a
	Ri	1.39±0.07a	0.60±0.04a	1.99±0.05a	0.45±0.06a	2.32±0.26a	0.23±0.03a
Significance							
Pb		0.00**	0.00**	0.00**	0.00**	0.00**	0.38ns
AMF		0.00**	0.00**	0.00**	0.00**	0.14ns	0.52ns
Pb × AMF		0.42ns	0.88ns	0.48ns	0.23ns	0.82ns	0.97ns

NM, non-inoculated control; Fm, inoculated with *F*. *mosseae*; and Ri, inoculated with *R*. *intraradices*. The results are the mean (n = 6) ± SD. The same letter within each Pb level indicates no significant difference (P < 0.05).

** P < 0.01; ns, no significance.

Two-way ANOVAs were used to determine the significance of the effects of Pb stress (Pb), AMF inoculation (AMF), and their interactions (Pb × AMF) on those parameters.

### Chlorophyll fluorescence and gas exchange

**Fig 3:**
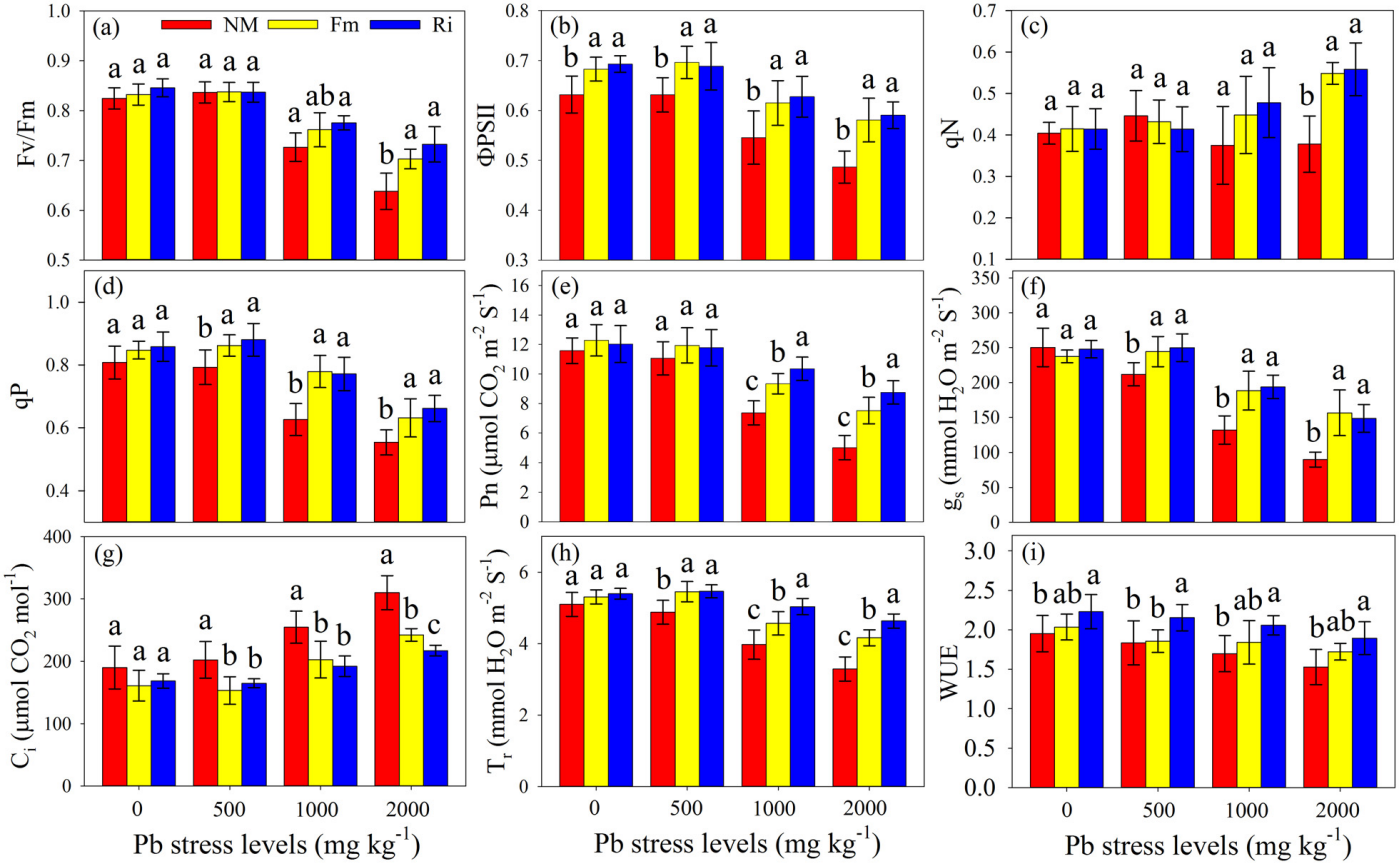
Fv/Fm (a), ΦPSII (b), qN (c), qP (d), Pn (e), g_s_ (f), C_i_ (g), T_r_ (h) and WUE (i) in *R*. *pseudoacacia* leaves were affected by Pb stress levels and AMF inoculation. NM, non-inoculated control; Fm, inoculated with *F*. *mosseae*; and Ri, inoculated with *R*. *intraradices*. The results are represented as the mean (n = 6) ± SD. The same letter indicates no significant difference among treatments (Duncan’s test, P < 0.05). Two-way ANOVAs were used to determine the significance of the effects of Pb stress (Pb), AMF inoculation (AMF), and their interactions (Pb × AMF) on those parameters, as shown in S2 and S3 Tables.

The leaf Fv/Fm, ΦPSII and qP of both mycorrhizal and non-mycorrhizal plants decreased with increasing Pb stress level, and the largest qN of mycorrhizal plants was found at the highest Pb stress level (Fig 3). Mycorrhizal plants had much higher Fv/Fm, ΦPSII and qP than those of non-mycorrhizal plants at 1000 and 2000 mg kg^-1^ Pb stress levels, although no difference could be found between Fm and Ri plants. In the control treatment (0 mg kg^-1^ Pb), the AMF inoculation did not show significant effects on chlorophyll florescence parameters except the ΦPSII, which were much higher in mycorrhizal plants than in non-mycorrhizal plants. The chlorophyll florescence parameters of Fm and Ri plants did not present differences in all the treatments.

The Leaf Pn, g_s_ and T_r_ decreased with increasing Pb stress levels, whereas Pb addition significantly increased the leaf C_i_, as shown in Fig 3. Mycorrhizal plants had much higher leaf Pn, g_s_ and T_r_ than non-mycorrhizal plants at 1000 and 2000 mg kg^-1^ Pb stress levels, but no difference in the g_s_ could be observed between Fm and Ri plants. By contrast, the plants that were inoculated with Ri had much higher Pn and T_r_ in comparison with these of plants that were inoculated with Fm in 1000 and 2000 mg kg^-1^ Pb treatments. In addition, the mycorrhizal plants had lower C_i_ than non-mycorrhizal plants in all the treatments, and the C_i_ of Ri plants was significantly lower compared to that of Fm plants, but only at the highest Pb stress level (2000 mg kg^-1^). Notably, the Ri plants had a significantly higher WUE value compared with that of non-mycorrhizal plants, but no significant difference in WUE could be detected between non-mycorrhizal and Fm plants in all the treatments (Fig 3).

### Enzyme activities of antioxidant systems

**Fig 4:**
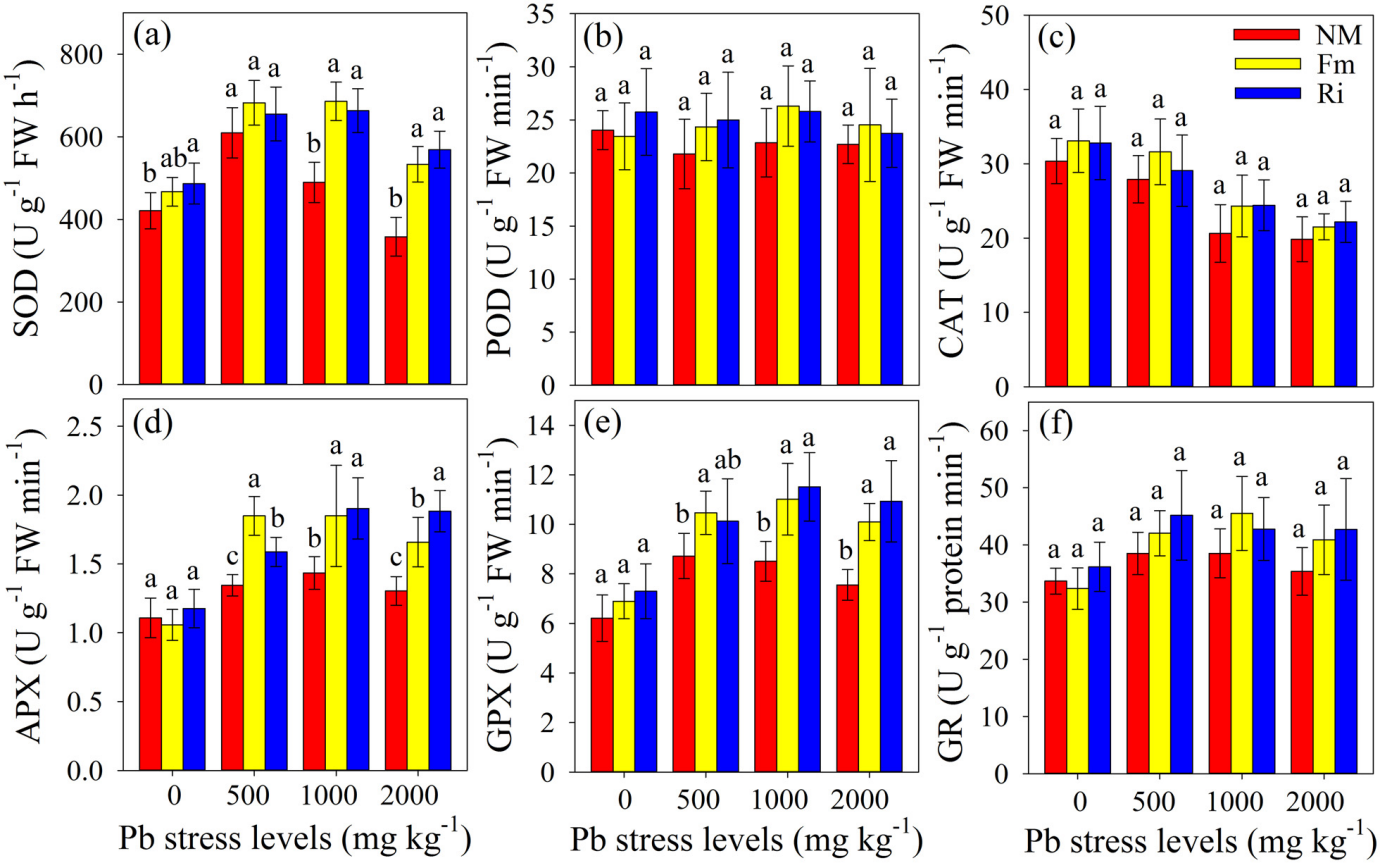
The SOD activity (a), POD activity (b), CAT activity (c), APX activity (d), GPX activity (e), and GR activity (f) in *R*. *pseudoacacia* leaves were affected by Pb stress levels and AMF inoculation. NM, non-inoculated control; Fm, inoculated with *F*. *mosseae*; and Ri, inoculated with *R*. *intraradices*. The results are shown as the mean (n = 6) ± SD. The same letter indicates no significant difference among treatments (Duncan’s test, P < 0.05). Two-way ANOVAs were used to determine the significance of the effects of the Pb level (Pb), AMF inoculation (AMF), and their interactions (Pb × AMF) on those parameters, as shown in S4 Table.

The activities of antioxidant enzymes in the leaves of mycorrhizal and non-mycorrhizal plants are shown in Fig 4. The SOD activity increased first and then decreased as Pb was added to the soil, and the highest value was found at the 500 mg kg^-1^ Pb stress level. Furthermore, the SOD activity of mycorrhizal plants was dramatically higher compared with that of non-mycorrhizal plants at the relatively high Pb stress levels (1000 and 2000 mg kg^-1^), but no difference could be found between Fm and Ri plants (Fig 4a). The APX and GPX activities in leaves from plants grown in Pb polluted soil were significant higher than those of the plants in the control treatment (0 mg kg^-1^), but no difference could be found among the plants at the 500, 1000 and 2000 mg kg^-1^ Pb stress levels (Fig 4d and 4e). Higher APX activity in leaves could be observed in the Fm plants than in the Ri plants at the 500 mg kg^-1^ Pb stress level, but the opposite result was detected in the 2000 mg kg^-1^ Pb treatment. Similarly, GR activity was induced by the low Pb stress level (500 mg kg^-1^), followed by a small decline at the highest Pb concentration (2000 mg kg^-1^), as shown in Fig 4f. However, no significant difference in GR activity could be observed among mycorrhizal and non-mycorrhizal plants. Interestingly, Pb addition and AMF inoculation had no effects on POD activity in the leaves of both mycorrhizal and non-mycorrhizal plants in all treatments (Fig 4b). The CAT activity greatly decreased with the increasing Pb stress level, but no significant difference could be found among mycorrhizal and non-mycorrhizal plants in all treatments (Fig 4c).

### Hydrogen peroxide and lipid peroxidation contents

**Fig 5:**
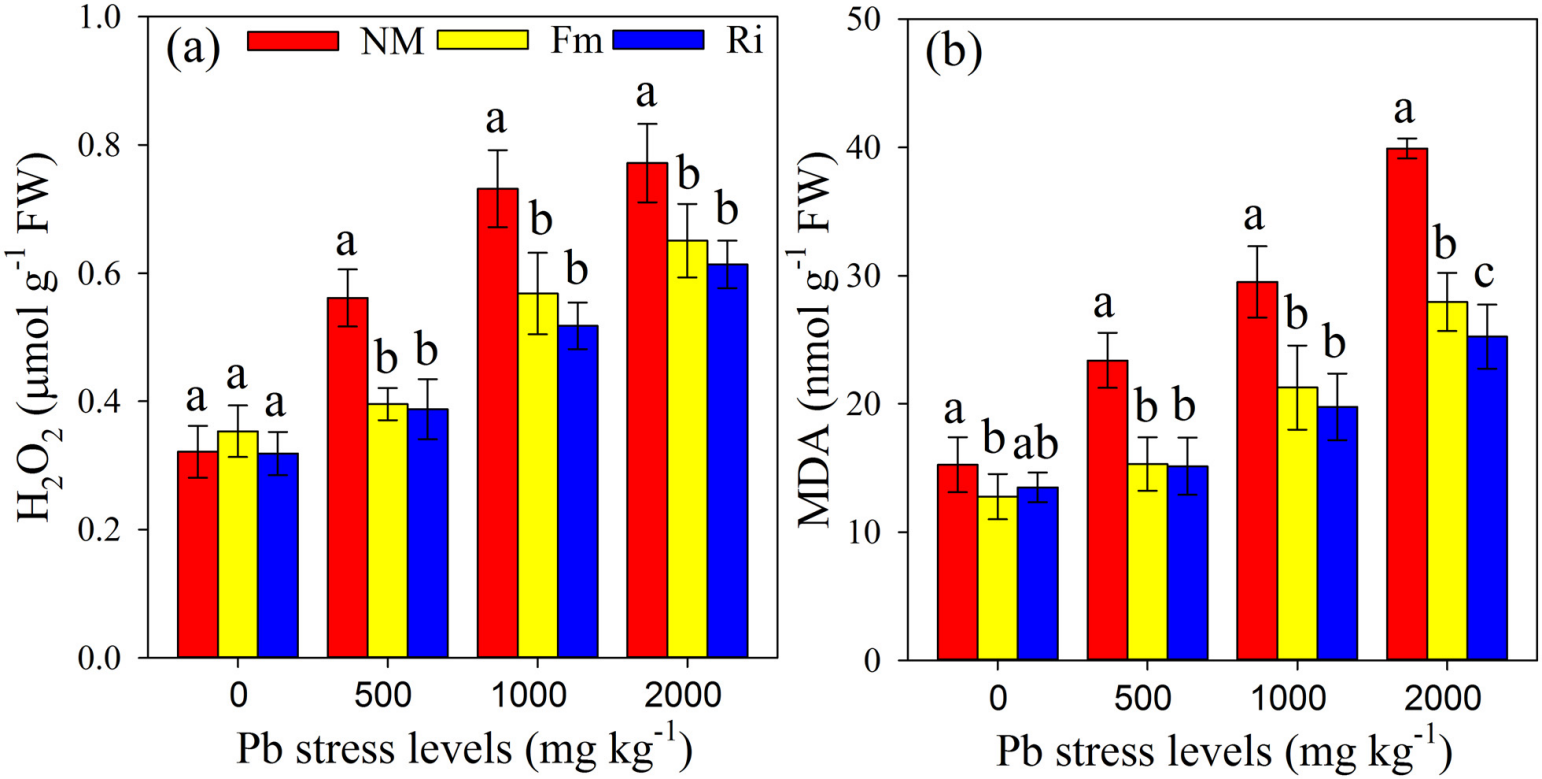
The H_2_O_2_ content (a) and MDA content (b) in *R*. *pseudoacacia* leaves were affected by Pb stress levels and AMF inoculation. NM, non-inoculated control; Fm, inoculated with *F*. *mosseae*; and Ri, inoculated with *R*. *intraradices*. The results are the mean (n = 6) ± SD. The same letter indicates no significant difference among treatments (Duncan’s test, P < 0.05). Two-way ANOVAs were used to determine the significance of the effects of the Pb level (Pb), AMF inoculation (AMF), and their interactions (Pb × AMF) on those parameters, as shown in S5 Table.

The H_2_O_2_ and MDA contents in the leaves increased linearly with increasing Pb concentration in the soil (Fig 5a and 5b). However, the increases in the H_2_O_2_ and MDA contents of mycorrhizal plants were significantly lower than those of non-mycorrhizal ones. In the 2000 mg kg^-1^ Pb treatment, the H_2_O_2_ and MDA contents of non-mycorrhizal plants increased by 143% and 166% respectively, in relation to the plants from the control soil, with increased H_2_O_2_ and MDA contents at 86% and 123% for Fm plants, and increases of 95% H_2_O_2_ and 89% MDA contents for the Ri plants.

## Discussion

### Pb contamination inhibited the MC of *R*. *pseudoacacia*

The beneficial effects of AMF on plant growth and HM tolerance have been widely suggested by various studies [11]. However, these effects have partly depended on the complex and not easily resolved plant-fungus-HM combination, and they were generally influenced by the soil conditions. Therefore, information on the status of AMF associated with *R*. *pseudoacacia* under Pb stress conditions is the foundation for further understanding the exact mechanisms of AMF that are involved in plant growth improvement and HM resistance. In the current study, the MC exhibited a significantly negative correlation with the soil Pb concentration (P = 0.00 for Fm plants; P = 0.01 for Ri plants), which was consistent with our previous field research [15]. Specifically, the MC of Fm and Ri plants at the 2000 mg kg^-1^ Pb stress level were 46% and 18% lower compared with the ones in the control treatment (0 mg kg^-1^) (Fig 1). The negative effects of HMs on MC have previously been described by Chen et al [30], who found the inhibition of mycorrhizal spore germination and extraradical hyphal growth under a stressful environment [31]. The results from this study showed that the leaf Pn of *R*. *pseudoacacia* significantly decreased in response to Pb stress (Fig 3). Bago et al [32] suggested that between 4% and 20% of the plant’s total photosynthetic products were sent to the AMF, which is a fundamental aspect of the symbiosis. Thus, the reduction in the host leaf Pn was likely another reason for MC inhibition with the decrease of nutrition that was supplied to the AMF under Pb-stressed conditions.

### *F*. *mosseae* was likely to be more sensitive to Pb than *R*. *intraradices*

HMs have been shown to reduce, delay, or even eliminate the MC of various plants [33]. However, in the present study, the AMF propagules never disappeared completely even in soils that were polluted by 2000 mg kg^-1^ Pb (S1 Fig), indicating that the *F*. *mosseae* and *R*. *intraradices* used in this study had high tolerance to Pb contamination and might develop their own strategies to survive in Pb-polluted soil. However, the two AMF species (*F*. *mosseae* and *R*. *intraradices*) presented different degrees of tolerance to Pb contamination (Fig 1). With the increasing of Pb stress level, the MC of Fm plants reached a maximum (57%) at 500 mg kg^-1^ Pb treatment and then declined to 27% at the highest Pb stress level (2000 mg kg^-1^), and the MC of the Ri plants was significantly higher than that of Fm plants in the 2000 mg kg^-1^ Pb treatment, suggesting that *R*. *intraradices* likely had a higher HM tolerance compared with *F*. *mosseae* in soil that was seriously polluted by Pb. However, after reviewing the literatures, we found that HMs have also been shown to have positive or neutral effects on the MC of plants [34, 35]. These inconsistent results may be a consequence of a wide range of factors such as the AMF species, Pb concentration, plant species, heavy metal speciation, stage of plant development and environmental conditions [36].

### Mycorrhizal plants retained more Pb in roots to protect leaf tissues from damage

There are conflicting results about the roles of AMF in HM accumulation and translocation in different host tissues. Some studies reported higher HM concentrations in mycorrhizal plants, sometimes even resulting in a toxic level [30, 37], whereas others found dramatically lower HM concentrations in mycorrhizal plants compared with non-mycorrhizal ones [38]. In the current study, we investigated the Pb accumulation in the roots and leaves of *R*. *pseudoacacia* when they were subjected to four levels of Pb concentrations. There were no differences in the leaf Pb concentrations between mycorrhizal and non-mycorrhizal plants from the 0 and 500 mg kg^-1^ Pb treatments. By contrast, mycorrhizal plants had a much lower leaf Pb concentration than non-mycorrhizal plants at relatively high Pb stress levels (1000 and 2000 mg kg^-1^), and Ri plants accumulated a significantly larger amount of Pb in their leaves than Fm plants at the highest Pb stress level (Fig 2a). Furthermore, higher Pb concentrations were detected in roots of mycorrhizal plants compared with those of non-mycorrhizal plants in all treatments except the control (Fig 2b). The TF of Pb in mycorrhizal plants significantly decreased with the increasing Pb concentration and was much lower than that of non-mycorrhizal plants from the 500, 1000 and 2000 mg kg^-1^ Pb treatments (Fig 2c). The results were consistent with a study conducted by Sudová and Vosátka [39], who reported that the roots of mycorrhizal maize accumulated a larger amount of Pb than those of non-mycorrhizal maize, and the Pb concentration in the leaves of mycorrhizal maize was significantly lower compared to that of non-mycorrhizal maize. Vogel-Mikuš et al [40] suggested a large amount of HMs could be concentrated in mycorrhizal structures, such as fungal mycelia and vesicle, thereby minimizing metal translocation from the root to the aerial parts of the plant. Göhre and Paszkowski [41] subsequently reported that the fungal vesicle structures were similar to plant and fungal vacuoles, which were involved in storing toxic compounds, and they provided an additional detoxification mechanism. This assumption was supported by our data and an increase in the number of fungal vesicles could be observed with the increasing Pb stress level (S1 Fig). Upon transmission electron microscope analysis, our previous study further confirmed the localization of Pb in AMF, including the hyphal cell wall, hyphal membrane, vesicular and vacuole membranes, etc. [37]. Furthermore, AMF symbiosis also induce the expression of the phytochelatin synthase gene (*PCS1*) in a leguminous plant (*Sophora viciifolia*) that was exposed to Pb pollution [42], indicating another mechanism for Pb immobilization in the roots of plants that were enhanced by AMF.

### AMF inoculation maintained Chl *a* and Car contents

Leaf chlorosis is one of the most commonly observed consequences of Pb toxicity [3]. In the present study, the Chl *a* and total Chl contents in both mycorrhizal and non-mycorrhizal plants significantly decreased in a dose-dependent fashion with increasing levels of Pb exposure (Table 2), which has been reported by several studies on other plant species [43]. The results could be partly related to the peroxidation of chloroplast membranes as mediated by Pb, leading to chlorophyll degradation and photosynthesis inhibition [44]. However, Singh et al [45] also attributed the loss of Chl content to the interaction of Pb with the -SH group of enzymes involved in chlorophyll biosynthesis. A slower degradation rate of Chl *b* than that of Chl *a* could be observed under Pb stress conditions, and mycorrhizal plants had much higher Chl *a* content than that of non-mycorrhizal plants at 1000 and 2000 mg kg^-1^ Pb stress levels, as shown in Table 2. These results suggested that the toxic effects of Pb on Chl *a* is greater than it is on Chl *b*. Chl *a* is known to be one of the most important pigments in photosynthesis, and therefore, the Chl *a* decrease can significantly inhibit the leaf photosynthetic rate. Thus, maintaining a high Chl *a* content might be one of the most important strategies for alleviating Pb toxicity in mycorrhizal plants. The Chl *a*/*b* ratio is an indicator of functional pigment equipment and of the light adaptation of the photosynthetic apparatus [46]. The leaf Chl *a*/*b* ratio of non-mycorrhizal plants significantly decreased with increasing Pb stress levels (Table 2), indicating a shift in the PSII/PSI ratio [47] and/or a modification in the antennae composition [48]. Car acts as a light-harvesting pigments and plays an important role in protecting chlorophyll and membranes from destruction by quenching triplet chlorophylls and removing oxygen from the excited chlorophyll-oxygen complex [49]. Therefore, a reduction in Car could result in serious consequence for chlorophyll pigments. In this study, the Car content was found to be less affected in mycorrhizal plants relative to non-mycorrhizal ones under Pb stress conditions (Table 2), indicating another possible mechanism of Pb detoxification caused by AMF symbiosis.

### AMF inoculation protected PSII reaction center

Chlorophyll a fluorescence is a powerful, non-invasive tool for studying the photosynthetic apparatus and recently, it has been widely used in physiological and eco-physiological studies [50]. The leaf Fv/Fm ratio is a useful measure of the maximum quantum efficiency of PSII primary photochemistry, which is considered a reliable diagnostic indicator of damage caused by a variety of environmental stress [51]. In this study, the Fv/Fm ratio in both mycorrhizal and non-mycorrhizal plants decreased with the increasing Pb stress level, but the Fv/Fm ratio of mycorrhizal plants declined less than that of non-mycorrhizal ones (Fig 3). In addition, the Fv/Fm ratio in the leaves of mycorrhizal plants was much higher compared with that of non-mycorrhizal ones at 1000 and 2000 mg kg^-1^ Pb stress levels, although no differences could be found between Fm and Ri plants (Fig 3a). Ralph and Burchett [52] reported that an increase in the F0 is always accompanied by a decrease in the chlorophyll content under HM stress conditions, suggesting the destruction and loss of the PSII reaction center or the disruption of electron transport from the collecting antennas to the reaction centers [53]. Relatively higher F0 and lower Fm values were detected in the leaves of non-mycorrhizal plants compared with mycorrhizal plants in the present study (S2 Table). Together with the higher ΦPSII and qP in mycorrhizal plants relative to those of non-mycorrhizal ones, we can conclude that AMF inoculation has the ability to alleviate the toxic effects of Pb on the PSII reaction center, through improving the maximum photochemistry efficiency and the photosynthetic capacity of the host (*R*. *pseudoacacia*). Furthermore, a significantly higher qN in mycorrhizal plants compared with that of non-mycorrhizal plants could be detected at the most toxic Pb stress level (Fig 3). The ΦPSII decreased as a consequence of the increase in qN, indicating that the plants dissipated light as heat, thereby protecting the leaf from light-induced damage [54]. These results may be partly supported by our previous study [17], which also revealed that the toxic influence of abiotic stress on the PSII reaction center could be mitigated by AMF symbiosis.

### AMF inoculation improved gas exchange capacity

Photosynthesis is an essential physico-chemical process for transducing light energy into chemical energy. This provides the energy for organic metabolism (growth and development) and the building blocks from which protein and fats are eventually synthesized [55]. A plant’s ability to maintain its photosynthetic rates under stressful condition might be an important indicator for assessing the ability of the plant to survive in HM polluted soil [56]. Pn is the most important driving force in plant growth and development, CO_2_ fixation and nitrate assimilation. Our studies showed that Pb had a negative effect on the leaf Pn, g_s_ and T_r_ of both mycorrhizal and non-mycorrhizal plants. The reduction in the Pn could be partly attributed to the negative effects of Pb on the activities of photosynthetic carbon reduction cycle enzymes [57] as well as the loss of photosynthetic pigments (Table 2). Mateos-Naranjo et al [58] suggested that the decrease in pigment content or the increase in its degradation consequently exposed adverse effects on photosynthetic electron transport and it led to a decline in photosynthetic function. Numerous studies have shown that mycorrhizal and non-mycorrhizal plants generally presented different gas exchange activities under non-stressed and environmental stress conditions [59]. In the current study, significantly higher leaf Pn, g_s_ and T_r_, but lower C_i_ could be observed in mycorrhizal plants compared with those of non-mycorrhizal ones (Fig 3), suggesting that AMF symbiosis was able to improve the gas exchange capacity of the host likely by maintaining open stomata, reducing stomatal resistances and increasing transpiration fluxes.

### Antioxidant enzymes differed in sensitivities to Pb exposure

As a redox-inert metal, Pb has been reported to cause oxidative damage from ROS production by disrupting the metabolic balance and inactivating antioxidant pools in many plant species [60]. The increased ROS levels likely resulted from Pb damage to chloroplasts and mitochondrial electrons transport chains, thereby leading to the breakdown of proteins by oxidative reactions or proteolytic activity [3]. The antioxidant enzymes and certain metabolites play an important role in scavenging ROS concentrations and in minimizing oxidative stress in plant cells [3]. Numerous studies have indicated that various antioxidant enzymes such as SOD, POD, CAT, APX, GPX and GR (Fig 5) are indispensable compounds for the cellular defense strategy towards excessive ROS production in plant cells [61]. The efficient functioning of SOD blocks $\left({\text{O}}_{2}^{\cdot -}\right)$ driven cell damage by converting it to H_2_O_2_, which is then decomposed to water and molecular oxygen through the action of enzymes (POD, APX, GPX and/or CAT) that work at different locations in the cell [62]. Among these antioxidant enzymes, only the activities of APX, GPX and GR in the leaves of mycorrhizal and non-mycorrhizal plants were significantly enhanced by Pb addition (Fig 4). Pb ions were able to increase the level of superoxide radicals through direct and/or indirect pathways, resulting in the expression of genes encoding APX, GPX and GR enzymes in the leaves of these plants [5]. However, a slight decrease in the APX, GPX and GR activities of non-mycorrhizal and Fm plants could be observed at the highest Pb stress level (2000 mg kg^-1^), which can be partly explained by the inactivation of enzymes resulting from excess H_2_O_2_ or metal binding to the active center [63]. SOD is considered the first defense against ROS because it acts on superoxide radicals, and it can catalyze the disproportionation of superoxide free radicals that are generated by the univalent reduction of molecular oxygen to H_2_O_2_ and O_2_ in the different cellular compartments of plant cells [6]. In this study, The SOD activity of non-mycorrhizal plants increased to its maximum in the 500 mg kg^-1^ Pb treatment, and then it significantly decreased at the highest Pb stress level (Fig 4a). The increased SOD activity in response to Pb stress was likely related to the de-novo synthesis of the enzymatic protein [3], whereas excess Pb ions likely bind to the reaction center of the SOD enzyme and thus inhibit its activity at the highest Pb level. CAT is universally present in the oxidoreductase of peroxisomes and mitochondria where it decomposes H_2_O_2_ to water and molecular oxygen. However, the CAT activity significantly decreased with the increased Pb stress level (Fig 4c), suggesting a possible delay in the removal of H_2_O_2_ and toxic peroxides mediated by CAT, and in turn might result in lipid peroxidation as caused by free radical in plant cells [64]. A similar result has been reported by other studies when using different plant species under various stress conditions [65]. The reduced activities of CAT at relatively high Pb (> 500 mg kg^-1^) might be attributed to (1) the inactivation of enzyme protein from excess ROS and/or Pb ions, (2) decreased enzyme synthesis, and/or (3) changes in the assembly of enzyme subunits [3]. POD is a vital alternative mode of H_2_O_2_ destruction and could be found throughout the cell with a much greater affinity to H_2_O_2_ than CAT [66]. HMs have been shown to have positive, negative or neutral effects on POD activity in the leaves of different plant species. Reddy et al [67] found that the POD activities of horsegram (*Macrotyloma uniflorum* (Lam.) Verdc.) and bengalgram (*Cicer arietinum* L.) in Pb-polluted soil were higher than they were in uncontaminated soil. Islam et al [68] reported that high Pb concentration significantly decreased the POD activities of the *Elsholtzia argyi* plant, and Shi et al [69] showed that the POD activity of safflower (*Carthamus tinctorius* L.) was not influenced by the presence of Cd in soil. The results from our study indicated that the POD activity was unchanged in both mycorrhizal and non-mycorrhizal plants when they were subjected to different Pb stress levels (Fig 4b), suggesting that different antioxidant enzymes showed different sensitivity to HM contamination. The POD of *R*. *pseudoacacia* was likely more insensitive to Pb ions related to the SOD and CAT in the current study.

### AMF inoculation enhanced SOD, APX and GPX activities

**Fig 6:**
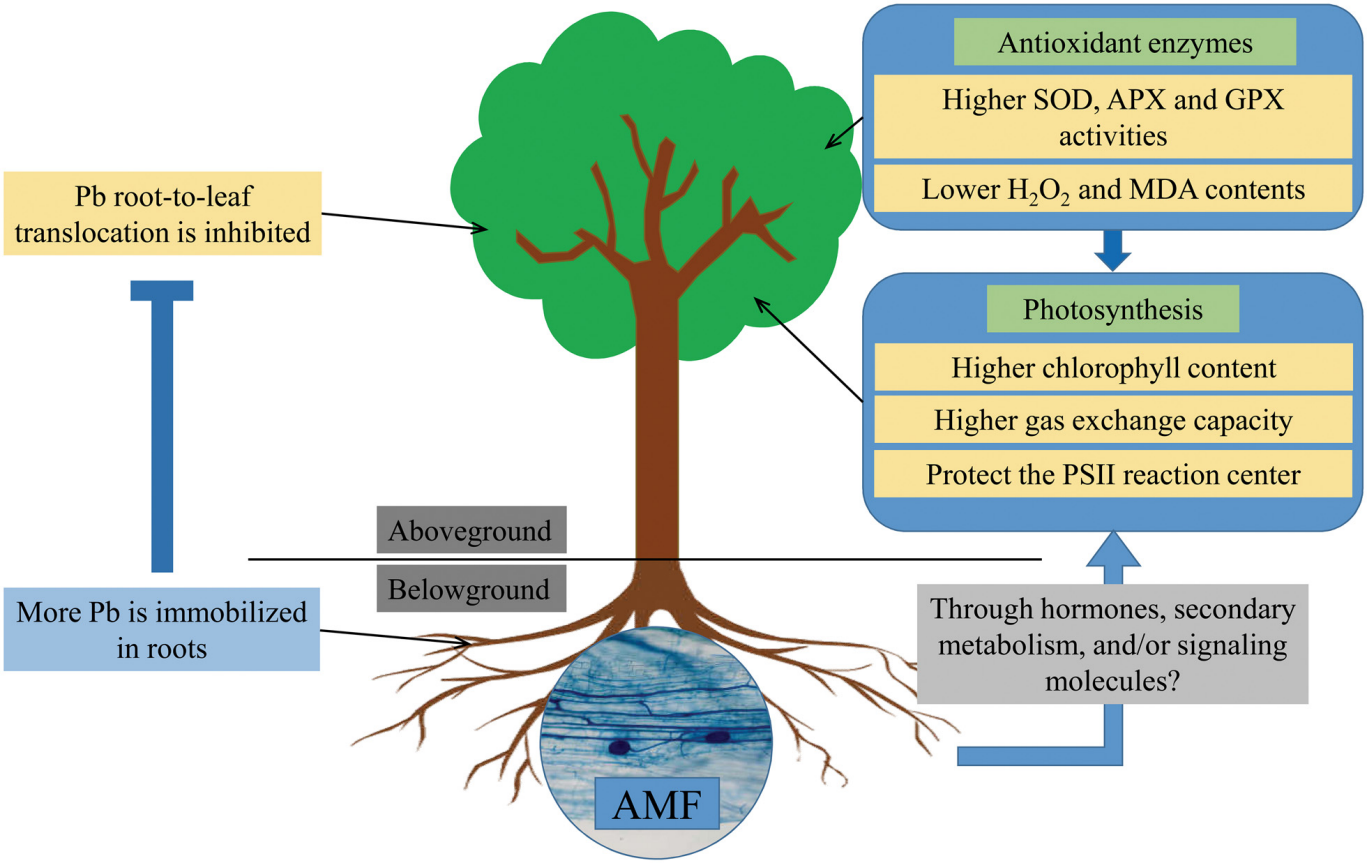
Roles of AMF inoculation in plant tolerance to Pb. AMF inoculation increased root Pb concentration but inhibited Pb root-to-leaf translocation. Mycorrhizal plants had higher leaf SOD, APX and GPX activities but lower leaf H_2_O_2_ and MDA contents compared with non-mycorrhizal plants. AMF inoculation increased Chl contents and gas exchange capacity, and protected the PSII reaction center under Pb stress conditions. AMF symbiosis causes physiological changes in plant aerial parts probably through affecting nutritional status, hormonal balance and/or secondary metabolism, and the translocation of small signaling molecules.

Numerous studies have reported that AMF can greatly improve plant growth and enhance tolerance towards biotic and abiotic stresses [14]. Latef and Chaoxing [70] indicated that the activation of the antioxidant capacity of mycorrhizal plants likely accounts for plant growth improvement and the alleviation of toxicity stress. However, our study confirmed that only certain species of enzymes (SOD, APX and GPX) were initiated by AMF symbiosis under Pb stress conditions (Figs 4 and 5). This phenomenon could be reasonably explained by Azcón et al [71], who suggested that fungal cells in symbiosis might primarily provide protection against HM-induced oxidative stress and the removal of ROS from plant cells [72]. The SOD activity in the leaves of mycorrhizal plants was significantly higher than that of non-mycorrhizal plants (Fig 4a) at 1000 and 2000 mg kg^-1^ Pb stress levels, indicating that superoxide radicals in mycorrhizal plant cells could be removed more effectively than they could be from non-mycorrhizal plants [73]. Together with the lower H_2_O_2_ and MDA contents detected in mycorrhizal plants relative to non-mycorrhizal plants, we can say that AMF symbiosis has the ability to protect plants against Pb pollution by enhancing SOD, APX and GPX activities in the leaves of *R*. *pseudoacacia* (Fig 6). However, it is important to note that no differences in the activities of POD, CAT and GR could be found between mycorrhizal and non-mycorrhizal plants in all treatments (Fig 4b, 4c and 4f). Therefore, more comprehensive studies are required to understand why AMF symbiosis only improved the activities of SOD, APX and GPX instead of POD, CAT and GR in the leaves of *R*. *pseudoacacia* under Pb stress conditions. Generally, APX and GPX are indispensable components of the ascorbate-glutathione (AsA-GSH) cycle, which plays an important role in scavenging reactive oxygen compounds in plants. APX is located in the cytosol, cell wall, vacuole and extracellular spaces, and it catalyzes the reduction of H_2_O_2_ to water by ascorbate. The resulting dehydroascorbate is reduced back to ascorbate with the help of GR [74]. GPX is located in the cytosol, cell wall, vacuole and extracellular spaces, where it consumes H_2_O_2_ to generate phenoxy compounds that are polymerized to produce cell wall components such as lignin [67]. In recent years, a growing number of studies have indicated that GPX functions in both hydroperoxide sensing and scavenging, and it also interacts specifically with protein partners to confer peroxide-induced oxidation [75]. In addition, Rahantaniaina et al [76] indicated that GPX might be a thioredoxin (TRX)-dependent peroxiredoxin, and it likely makes some contribution to the GSH oxidation, although it is not a strong candidate to account for GSSG accumulation during stress conditions. Thus, we can conclude that AMF symbiosis helps plants to scavenge ROS and maintain antioxidant level in the leaves of *R*. *pseudoacacia* under Pb stress conditions. This actions are likely achieved through the AsA-GSH cycle by involving various antioxidative enzymes and electron donors. However, detailed information about the relations among AMF and the metabolic pathways of SOD, APX and GPX in the leaves of *R*. *pseudoacacia* are still poorly understood, which requires further research. Moreover, it is necessary to keep in mind that other antioxidant enzymes and/or non-enzymatic antioxidants might also be involved and should be considered for future study.

### AMF inoculation reduced H_2_O_2_ and MDA contents

The H_2_O_2_ and MDA contents have been widely used as criteria for assessing HM injury in various plant species. Elevated H_2_O_2_ and MDA contents were observed in both mycorrhizal and non-mycorrhizal plants under Pb stress conditions, indicating that *R*. *pseudoacacia* was susceptible to oxidation from Pb toxicity (Fig 5a and 5b). This result was consistent with the findings of Sun et al [77], who reported increased H_2_O_2_ content in *Hypnum plumaeforme* with increasing soil Pb concentration. Pb induced the loss of membrane permeability coupled with the increasing MDA production observed in *Vallisneria natans* [78], which might have resulted from the repetitive formation of short-chain alkanes and lipid acid aldehydes caused by Pb ions. However, in the present study, mycorrhizal plants had significantly lower H_2_O_2_ and MDA contents than non-mycorrhizal ones, indicating that AMF symbiosis could help plants to reduce the oxidative damage caused by Pb contamination. Our data then suggested that the positive effects of AM colonization on the plant antioxidant system might have contributed to the enhancement of SOD, APX and GPX activities in the *R*. *pseudoacacia* leaves. However, the increased activities of these antioxidant enzymes (SOD, APX and GPX) in mycorrhizal plants did not provide enough protection against ROS as judged by the simultaneously exaggerated oxidative stress (H_2_O_2_ and MDA contents). Therefore, an enhanced antioxidative metabolism could increase the capacity of mycorrhizal plants to scavenge ROS only to a certain extent [79].

## Conclusions

As noted above, the symbiosis between a leguminous tree (*R*. *pseudoacacia*) and two AMF species (*F*. *mosseae* and *R*. *intraradices*) were well established under Pb stress conditions (S1 Fig). Both *F*. *mosseae* and *R*. *intraradices* inoculations could decrease Pb root-to-leaf translocation and thus protect the aerial parts of plants from damage. Additionally, AMF increased not only growth but alsophotosynthetic pigment content, leaf photosynthesis, and antioxidant enzyme activities of *R*. *pseudoacacia*, suggesting that AMF symbiosis could help host plant to cope with Pb toxicity. The lower H_2_O_2_ and MDA contents are associated with the protection of the photosynthetic apparatus of the mycorrhizal plants could be also observed in the current study (Fig 5). A model summarizing the roles of AMF inoculation in plant tolerance to Pb is presented in Fig 6. The effects of AMF symbiosis on physiological changes of plant aerial parts likely to be the results of different nutritional status, the alteration of hormonal balance and/or secondary metabolism, and the translocation of small signaling molecules [80], which needs to be further investigated. In addition, further studies are still required to reveal the interactions between AMF and indigenous soil microorganisms in phytostabilization and ecological stability in larger scales and under more complex environmental conditions.

## Supporting Information

S1 Fig Hyphae and vesicle structures of *R*. *intraradices* colonized in *R*. *pseudoacacia* roots in the presence of 0 mg kg^-1^ (a), 500 mg kg^-1^ (b), 1000 mg kg^-1^ (c) and 2000 mg kg^-1^ (d) Pb in soil. (DOCX)

S2 Fig Mycorrhizal dependency of *R*. *pseudoacacia* subjected to four Pb stress levels. (DOCX)

S1 Table Multiple ANOVA comparisons of MC, Pb concentration in the leaves and roots, and the TF value of *R*. *pseudoacacia* under Pb stress and AMF inoculation treatments. (DOCX)

S2 Table Multiple ANOVA comparisons of Fv/Fm, ΦPSII, qN and qP in *R*. *pseudoacacia* leaves under Pb stress and AMF inoculation treatments. (DOCX)

S3 Table Multiple ANOVA comparisons of Pn, g_s_, C_i_, T_r_ and WUE in *R*. *pseudoacacia* leaves under Pb stress and AMF inoculation treatments. (DOCX)

S4 Table Multiple ANOVA comparisons of SOD, POD, CAT, APX, GPX and GR activities in *R*. *pseudoacacia* leaves under Pb stress and AMF inoculation treatments. (DOCX)

S5 Table Multiple ANOVA comparisons of H_2_O_2_ and MDA contents in *R*. *pseudoacacia* leaves under Pb stress and AMF inoculation treatments. (DOCX)
