# Supplementary material for: The Combined Effects of Arbuscular Mycorrhizal Fungi (AMF) and Lead (Pb) Stress on Pb Accumulation, Plant Growth Parameters, Photosynthesis, and Antioxidant Enzymes in Robinia pseudoacacia L
Source: PLoS One. 2015 Dec 23;10(12):e0145726. doi: 10.1371/journal.pone.0145726 (PMC4689355; doi:10.1371/journal.pone.0145726)
Supplement: S1 Fig — (DOCX) [file pone.0145726.s001.docx]

**
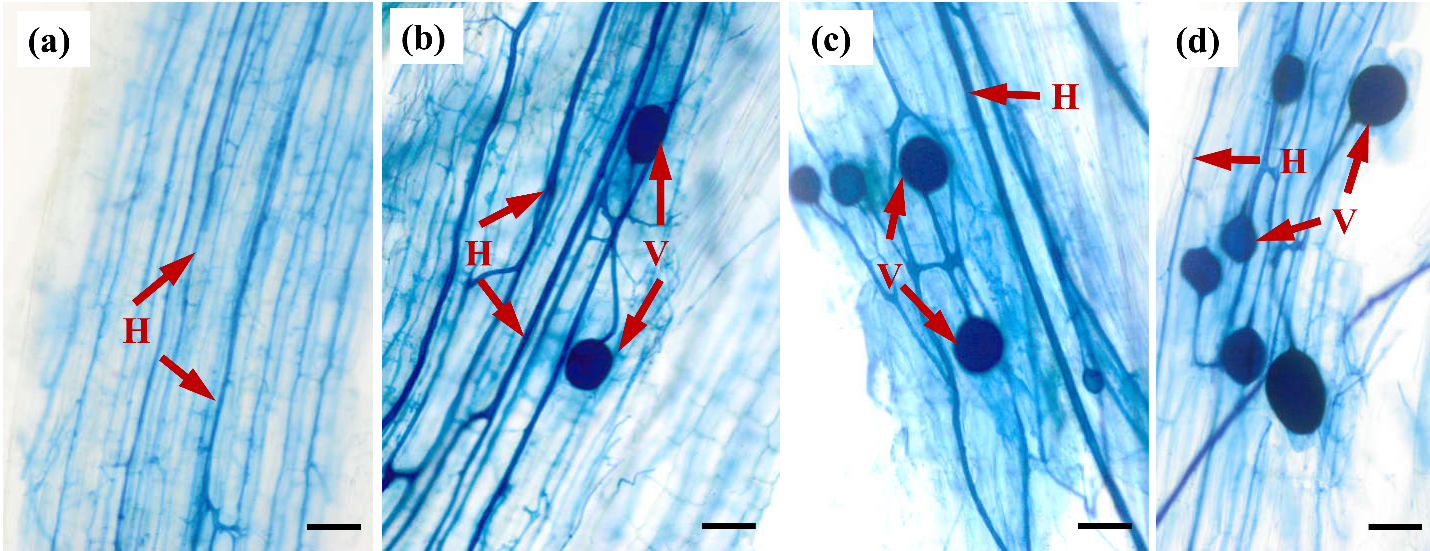
**

**S1 Fig.** **Hyphae and vesicle structures of *R. intraradices* colonized in roots of *R. pseudoacacia* in the presence of 0 mg kg^-1^ (a), 500 mg kg^-1^ (b), 1000 mg kg^-1^ (c) and 2000 mg kg^-1^ (d) Pb in soil.** H, hyphae. V, vesicles. Bars present the length of 50 μm for all images.
