# Supplementary material for: The Combined Effects of Arbuscular Mycorrhizal Fungi (AMF) and Lead (Pb) Stress on Pb Accumulation, Plant Growth Parameters, Photosynthesis, and Antioxidant Enzymes in Robinia pseudoacacia L
Source: PLoS One. 2015 Dec 23;10(12):e0145726. doi: 10.1371/journal.pone.0145726 (PMC4689355; doi:10.1371/journal.pone.0145726)
Supplement: S2 Fig — (DOCX) [file pone.0145726.s002.docx]

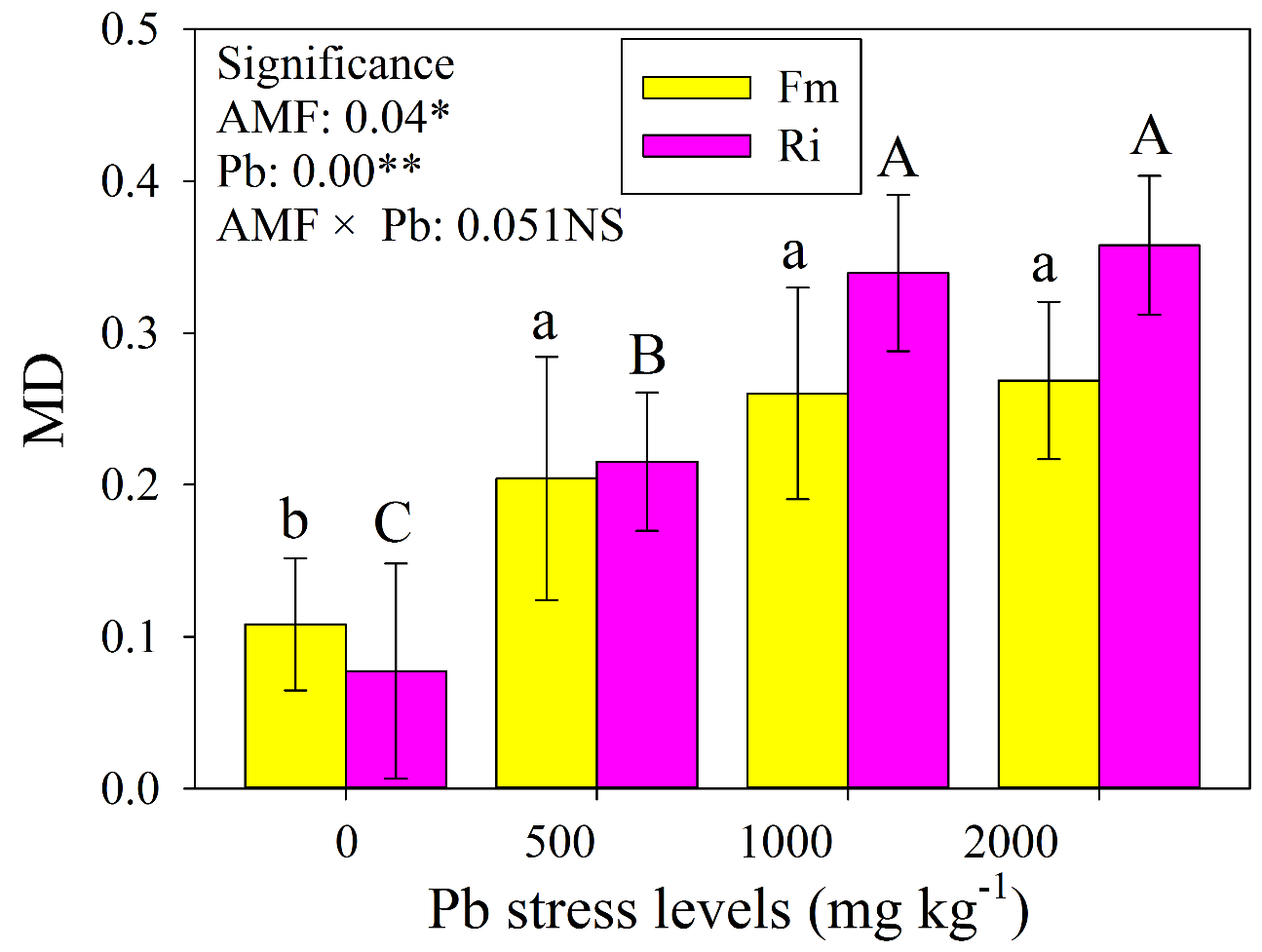


**S2** **Fig. Mycorrhizal dependency of *R. pseudoacacia* subjected to four Pb stress levels.** Fm, inoculated with *F*. *mosseae*; and Ri, inoculated with *R*. *intraradices*. Values are means ± SD of six replicates. The means marked by the same letters are not significantly different according to Duncan's multiple range tests at the level P < 0.05. ** P < 0.01; * P < 0.05; NS, no significance.
