# Supplementary material for: The Combined Effects of Arbuscular Mycorrhizal Fungi (AMF) and Lead (Pb) Stress on Pb Accumulation, Plant Growth Parameters, Photosynthesis, and Antioxidant Enzymes in Robinia pseudoacacia L
Source: PLoS One. 2015 Dec 23;10(12):e0145726. doi: 10.1371/journal.pone.0145726 (PMC4689355; doi:10.1371/journal.pone.0145726)
Supplement: S1 Table — (DOCX) [file pone.0145726.s003.docx]

**S1 Table**. **Multiple ANOVA comparisons of MC, Pb concentration in the leaves and roots, and the TF value of *R. pseudoacacia* under Pb stress and AMF inoculation treatments.**

| **Pb level (mg kg^-1^)** | **AMF inoculation** | **MC** | **Pb concentration (mg kg^-1^)** | | **TF** |
| --- | --- | --- | --- | --- | --- |
|  |  |  | **Leaf** | **Root** |  |
| 0 | NM | - | 9.65±2.46a | 11.1±1.99b | 0.87±0.19a |
|  | Fm | 49.8±5.69b | 8.88±2.05a | 11.0±1.95b | 0.84±0.28a |
|  | Ri | 45.7±5.24a | 11.1±2.30a | 13.2±0.89a | 0.84±0.17a |
| 500 | NM | - | 88.4±9.96a | 125±14.6b | 0.71±0.09a |
|  | Fm | 57.2±5.01a | 105±25.5a | 208±26.7a | 0.51±0.13b |
|  | Ri | 43.7±4.33a | 109±13.3a | 223±26.0a | 0.49±0.10b |
| 1000 | NM | - | 138±14.1a | 189±9.69b | 0.73±0.08a |
|  | Fm | 39.9±2.89c | 118±11.9b | 317±22.2a | 0.37±0.05b |
|  | Ri | 45.0±7.15a | 115±7.93b | 347±39.9a | 0.33±0.04b |
| 2000 | NM | - | 218±16.3a | 390±18.3c | 0.56±0.04a |
|  | Fm | 26.9±3.23d | 155±17.1b | 541±53.0b | 0.29±0.05b |
|  | Ri | 37.2±2.92b | 131±14.5c | 604±63.6a | 0.22±0.01c |
| Significance | |  |  |  |  |
| Pb | | 0.00** | 0.00** | 0.00** | 0.00** |
| AMF | | 0.00** | 0.00** | 0.00** | 0.00** |
| Pb × AMF | | 0.00** | 0.00** | 0.00** | 0.02* |

NM, non-inoculated control; Fm, inoculated with *F*. *mosseae*; and Ri, inoculated with *R*. *intraradices*. Each value is the mean (±SD) of six replicates (Duncan’s test, P < 0.05). The same letter within each Pb level indicates no significance (P < 0.05). MC, mycorrhizal colonization. ** P < 0.01; * P < 0.05.
