# Supplementary material for: The Combined Effects of Arbuscular Mycorrhizal Fungi (AMF) and Lead (Pb) Stress on Pb Accumulation, Plant Growth Parameters, Photosynthesis, and Antioxidant Enzymes in Robinia pseudoacacia L
Source: PLoS One. 2015 Dec 23;10(12):e0145726. doi: 10.1371/journal.pone.0145726 (PMC4689355; doi:10.1371/journal.pone.0145726)
Supplement: S2 Table — (DOCX) [file pone.0145726.s004.docx]

**S2 Table**. **Multiple ANOVA comparisons of Fv/Fm, ΦPSII, qN and qP in *R. pseudoacacia* leaves under Pb stress and AMF inoculation treatments.**

| **Pb level (mg kg^-1^)** | **AMF inoculation** | **F0** | **Fm** | **Fv/Fm** | **ΦPSII** | **qN** | **qP** |
| --- | --- | --- | --- | --- | --- | --- | --- |
| 0 | NM | 0.09±0.01a | 0.51±0.01b | 0.82±0.02a | 0.63±0.04b | 0.40±0.03a | 0.81±0.05a |
|  | Fm | 0.08±0.01a | 0.51±0.02b | 0.83±0.02a | 0.68±0.02a | 0.41±0.05a | 0.85±0.03a |
|  | Ri | 0.08±0.01a | 0.54±0.03a | 0.85±0.02a | 0.069±0.02a | 0.41±0.05a | 0.86±0.05a |
| 500 | NM | 0.09±0.01a | 0.53±0.01a | 0.84±0.02a | 0.63±0.03b | 0.45±0.06a | 0.79±0.05b |
|  | Fm | 0.08±0.01a | 0.51±0.01a | 0.84±0.02a | 0.70±0.03a | 0.43±0.05a | 0.86±0.03a |
|  | Ri | 0.08±0.00a | 0.49±0.02b | 0.84±0.02a | 0.69±0.05a | 0.41±0.05a | 0.88±0.05a |
| 1000 | NM | 0.12±0.01a | 0.42±0.01a | 0.73±0.03b | 0.55±0.05b | 0.37±0.09a | 0.63±0.05b |
|  | Fm | 0.11±0.01ab | 0.45±0.02a | 0.76±0.03ab | 0.61±0.04a | 0.45±0.09a | 0.78±0.05a |
|  | Ri | 0.10±0.01b | 0.44±0.03a | 0.78±0.01a | 0.63±0.04a | 0.48±0.08a | 0.77±0.05a |
| 2000 | NM | 0.12±0.01a | 0.34±0.01b | 0.64±0.04b | 0.49±0.03b | 0.38±0.07b | 0.55±0.04b |
|  | Fm | 0.11±0.01ab | 0.37±0.03a | 0.70±0.02a | 0.58±0.04a | 0.55±0.03a | 0.63±0.06a |
|  | Ri | 0.10±0.01b | 0.38±0.02a | 0.73±0.04a | 0.59±0.03a | 0.56±0.06a | 0.66±0.04a |
| Significance | |  |  |  |  |  |  |
| Pb | | 0.00** | 0.00** | 0.00** | 0.00** | 0.00** | 0.00** |
| AMF | | 0.00** | 0.06NS | 0.00** | 0.00** | 0.00** | 0.00** |
| Pb × AMF | | 0.45NS | 0.00** | 0.00** | 0.71NS | 0.00** | 0.12NS |

NM, non-inoculated control; Fm, inoculated with *F*. *mosseae*; and Ri, inoculated with *R*. *intraradices*. Each value is the mean (±SD) of six replicates (Duncan’s test, P < 0.05). The same letter within each Pb level indicates no significant difference (P < 0.05). ** P < 0.01; NS, no significance.
