# Supplementary material for: The Combined Effects of Arbuscular Mycorrhizal Fungi (AMF) and Lead (Pb) Stress on Pb Accumulation, Plant Growth Parameters, Photosynthesis, and Antioxidant Enzymes in Robinia pseudoacacia L
Source: PLoS One. 2015 Dec 23;10(12):e0145726. doi: 10.1371/journal.pone.0145726 (PMC4689355; doi:10.1371/journal.pone.0145726)
Supplement: S3 Table — (DOCX) [file pone.0145726.s005.docx]

**S3 Table**. **Multiple ANOVA comparisons of Pn, gs, Ci, Tr and WUE in *R. pseudoacacia* leaves under Pb stress and AMF inoculation treatments.**

| **Pb level (mg kg^-1^)** | **AMF inoculation** | **Pn (µmol CO_2_ m^-2^ S^-1^)** | **g_s_ (mmol H_2_O m^-2^ S^-1^)** | **C_i_ (µmol CO_2_ mol^-1^)** | **T_r_ (mmol H_2_O m^-2^ S^-1^)** | **WUE** |
| --- | --- | --- | --- | --- | --- | --- |
| 0 | NM | 11.6±0.87a | 250±27.7a | 190±34.4a | 5.30±0.34a | 1.95±0.23b |
|  | Fm | 12.3±1.06a | 237±9.09a | 161±24.6a | 5.31±0.20a | 2.03±0.16ab |
|  | Ri | 12.0±1.25a | 248±12.6a | 168±11.4a | 5.40±0.15a | 2.23±0.22a |
| 500 | NM | 11.1±1.12a | 212±16.6b | 202±29.3a | 4.88±0.33b | 1.83±0.28b |
|  | Fm | 11.9±1.19a | 244±21.7a | 153±22.1b | 5.45±0.28a | 1.86±0.14b |
|  | Ri | 11.8±1.23a | 250±19.9a | 165±6.96b | 5.46±0.18a | 2.15±0.17a |
| 1000 | NM | 7.36±0.82c | 132±20.4b | 255±25.8a | 3.97±0.41c | 1.70±0.23b |
|  | Fm | 9.34±0.68b | 188±27.8a | 203±29.4b | 4.57±0.33b | 1.84±0.27ab |
|  | Ri | 10.36±0.79a | 194±16.7a | 192±16.4b | 5.04±0.23a | 2.06±0.12a |
| 2000 | NM | 5.02±0.81c | 89.8±10.7b | 310±27.3a | 3.29±0.34c | 1.53±0.22b |
|  | Fm | 7.52±0.90b | 156±32.9a | 242±10.1b | 4.16±0.22b | 1.72±0.11ab |
|  | Ri | 8.76±0.79a | 148±20.1a | 217±8.72c | 4.63±0.20a | 1.89±0.21a |
| Significance | |  |  |  |  |  |
| Pb | | 0.00** | 0.00** | 0.00** | 0.00** | 0.00** |
| AMF | | 0.00** | 0.00** | 0.00** | 0.00** | 0.00** |
| Pb × AMF | | 0.00** | 0.00** | 0.012* | 0.00** | 0.96NS |

NM, non-inoculated control; Fm, inoculated with *F*. *mosseae*; and Ri, inoculated with *R*. *intraradices*. Each value is the mean (±SD) of six replicates (Duncan’s test, P < 0.05). The same letter within each Pb level indicates no significant difference (P < 0.05). ** P < 0.01; * P < 0.05; NS, no significane.
