# Supplementary material for: The Combined Effects of Arbuscular Mycorrhizal Fungi (AMF) and Lead (Pb) Stress on Pb Accumulation, Plant Growth Parameters, Photosynthesis, and Antioxidant Enzymes in Robinia pseudoacacia L
Source: PLoS One. 2015 Dec 23;10(12):e0145726. doi: 10.1371/journal.pone.0145726 (PMC4689355; doi:10.1371/journal.pone.0145726)
Supplement: S4 Table — (DOCX) [file pone.0145726.s006.docx]

**S4 Table**. **Multiple ANOVA comparisons of SOD, POD, CAT, APX, GPX and GR activities in *R. pseudoacacia* leaves under Pb stress and AMF inoculation treatments.**

| **Pb level (mg kg^-1^)** | **AMF inoculation** | **SOD (U g^-1^ FW h^-1^)** | **POD (U g^-1^ FW min^-1^)** | **CAT (U g^-1^ FW min^-1^)** | **APX (U g^-1^ FW min^-1^)** | **GPX (U g^-1^ FW min^-1^)** | **GR (U g^-1^ protein min^-1^)** |
| --- | --- | --- | --- | --- | --- | --- | --- |
| 0 | NM | 421±43.5b | 24.0±1.83a | 30.4±3.05a | 1.11±0.14a | 6.21±0.94a | 33.7±2.27a |
|  | Fm | 467±34.9ab | 23.4±3.14a | 33.1±4.26a | 1.06±0.11a | 6.89±0.71a | 32.4±3.62a |
|  | Ri | 487±49.4a | 25.8±4.07a | 32.8±4.94a | 1.18±0.14a | 7.30±1.11a | 36.18±4.33a |
| 500 | NM | 610±60.9a | 21.8±3.27a | 27.9±3.17a | 1.34±0.08c | 8.72±0.92b | 38.5±3.71a |
|  | Fm | 682±54.0a | 24.3±3.17a | 31.6±4.41a | 1.85±0.14a | 10.5±0.88a | 42.1±3.95a |
|  | Ri | 655±65.3a | 25.0±4.51a | 29.1±4.79a | 1.59±0.11b | 10.1±1.71ab | 45.2±7.84a |
| 1000 | NM | 490±48.8b | 22.9±3.22a | 20.6±3.88a | 1.43±0.12b | 8.51±0.81b | 38.6±4.27a |
|  | Fm | 686±47.5a | 26.3±3.79a | 24.3±4.15a | 1.85±0.37a | 11.0±1.45a | 45.5±6.46a |
|  | Ri | 663±53.2a | 25.8±3.87a | 24.4±3.42a | 1.90±0.22a | 11.5±1.38a | 42.8±5.50a |
| 2000 | NM | 358±46.9b | 22.7±1.82a | 19.9±3.01a | 1.30±0.10c | 7.56±0.62b | 35.4±4.17a |
|  | Fm | 534±43.0a | 24.5±5.33a | 21.5±1.75a | 1.66±0.18b | 10.1±0.75a | 40.9±6.08a |
|  | Ri | 569±45.0a | 23.7±3.21a | 22.2±2.77a | 1.88±0.15a | 10.9±1.64a | 42.72±8.90a |
| Significance | |  |  |  |  |  |  |
| Pb | | 0.00** | 0.67NS | 0.00** | 0.00** | 0.00** | 0.00** |
| AMF | | 0.00** | 0.07NS | 0.02* | 0.00** | 0.005** | 0.00** |
| Pb × AMF | | 0.00** | 0.81NS | 0.94NS | 0.00** | 0.50NS | 0.00** |

NM, non-inoculated control; Fm, inoculated with *F*. *mosseae*; and Ri, inoculated with *R*. *intraradices*. Each value is the mean (±SD) of six replicates (Duncan’s test, P < 0.05). The same letter within each Pb level indicates no significant difference (P < 0.05). ** P < 0.01; * P < 0.05; NS, no significance.
