# Supplementary material for: The Combined Effects of Arbuscular Mycorrhizal Fungi (AMF) and Lead (Pb) Stress on Pb Accumulation, Plant Growth Parameters, Photosynthesis, and Antioxidant Enzymes in Robinia pseudoacacia L
Source: PLoS One. 2015 Dec 23;10(12):e0145726. doi: 10.1371/journal.pone.0145726 (PMC4689355; doi:10.1371/journal.pone.0145726)
Supplement: S5 Table — (DOCX) [file pone.0145726.s007.docx]

**S5 Table**. **Multiple ANOVA comparisons of H_2_O_2_ and MDA contents in *R. pseudoacacia* leaves under Pb stress and AMF inoculation treatments.**

| **Pb level (mg kg^-1^)** | **AMF inoculation** | **H_2_O_2_ content (µmol g^-1^ FW)** | **MDA content (nmol g^-1^ FW)** |
| --- | --- | --- | --- |
| 0 | NM | 0.32±0.04a | 15.3±2.14a |
|  | Fm | 0.35±0.04a | 12.8±1.75b |
|  | Ri | 0.32±0.03a | 13.5±1.15ab |
| 500 | NM | 0.56±0.04a | 23.4±2.16a |
|  | Fm | 0.40±0.03b | 15.3±2.09b |
|  | Ri | 0.39±0.05b | 15.1±2.23b |
| 1000 | NM | 0.73±0.06a | 29.5±2.78a |
|  | Fm | 0.57±0.06b | 21.3±3.28b |
|  | Ri | 0.52±0.04b | 19.8±2.60b |
| 2000 | NM | 0.77±0.06a | 39.9±0.78a |
|  | Fm | 0.65±0.06b | 28.0±2.27b |
|  | Ri | 0.61±0.04b | 25.3±2.50c |
| Significance | |  |  |
| Pb | | 0.00** | 0.00** |
| AMF | | 0.00** | 0.00** |
| Pb × AMF | | 0.04* | 0.00** |

NM, non-inoculated control; Fm, inoculated with *F*. *mosseae*; and Ri, inoculated with *R*. *intraradices*. Each value is the mean (±SD) of six replicates (Duncan’s test, P < 0.05). The same letter within each Pb level indicates no significant difference (P < 0.05). ** P < 0.01; * P < 0.05.
